# Nicotinamide mononucleotide restores impaired metabolism, endothelial cell proliferation and angiogenesis in old sedentary male mice

**DOI:** 10.1016/j.isci.2024.111656

**Published:** 2024-12-20

**Authors:** Kevin Kiesworo, Thomas Agius, Michael R. Macarthur, Martine Lambelet, Arnaud Lyon, Jing Zhang, Guillermo Turiel, Zheng Fan, Sènan d’Almeida, Korkut Uygun, Heidi Yeh, Sébastien Déglise, Katrien de Bock, Sarah J. Mitchell, Alejandro Ocampo, Florent Allagnat, Alban Longchamp

**Affiliations:** 1 Department of Vascular Surgery, Lausanne University Hospital (CHUV), Lausanne, Switzerland; 2 Transplant Center, Department of Surgery, Massachusetts General Hospital, Harvard Medical School, Boston, MA, USA; 3 Center for Engineering in Medicine, Department of Surgery, Massachusetts General Hospital, Harvard Medical School, Boston, MA, USA; 4 Lewis-Sigler Institute for Integrative Genomics, Princeton University, Princeton, NJ, USA; 5 Ludwig Princeton Branch, Princeton University, Princeton, NJ, USA; 6 Transplantation Centre and Transplantation Immunopathology Laboratory, Department of Medicine, Lausanne University Hospital (CHUV), Lausanne, Switzerland; 7 Department of Health Sciences and Technology, ETH Zurich, Zurich, Switzerland; 8 Flow Cytometry Core Facility, EPFL, Lausanne, Switzerland; 9 Department of Biomedical Sciences, Lausanne University (UNIL), Lausanne, Switzerland

**Keywords:** Cellular physiology, Metabolomics

## Abstract

Aging is accompanied by a decline in neovascularization potential and increased susceptibility to ischemic injury. Here, we confirm the age-related impaired neovascularization following ischemic leg injury and impaired angiogenesis. The age-related deficits in angiogenesis arose primarily from diminished EC proliferation capacity, but not migration or VEGF sensitivity. Aged EC harvested from the mouse skeletal muscle displayed a pro-angiogenic gene expression phenotype, along with considerable changes in metabolic genes. Metabolomics analysis and ^13^C glucose tracing revealed impaired ATP production and blockade in glycolysis and TCA cycle in late passage HUVECs, which occurred at nicotinamide adenine dinucleotide (NAD⁺)-dependent steps, along with NAD^+^ depletion. Supplementation with nicotinamide mononucleotide (NMN), a precursor of NAD⁺, enhances late-passage EC proliferation and sprouting angiogenesis from aged mice aortas. Taken together, our study illustrates the importance of NAD^+^-dependent metabolism in the maintenance of EC proliferation capacity with age, and the therapeutic potential of NAD precursors.

## Highlights

• Pro-angiogenic genes expression is increased in aged endothelial cells (ECs)
• Age-associated defect in angiogenesis is a result of impaired EC proliferation
• Aged ECs display altered glycolysis and TCA cycle activity at NAD⁺-dependent steps
• Supplementation with NAD^+^ precursor enhances EC proliferation and angiogenesis

## Graphical abstract

**Figure:**
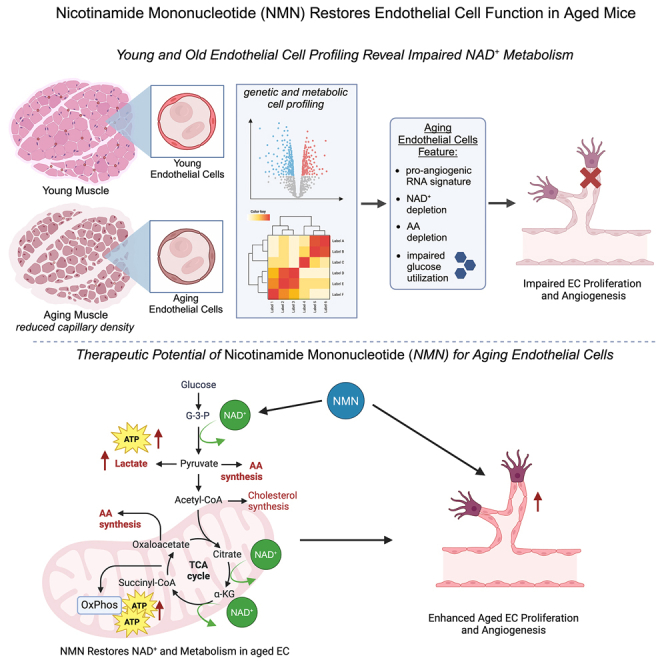

## Introduction

Aging is a progressive process characterized by a decrease in tissue and cell functions and an increased incidence of age-related diseases, such as neurodegenerative diseases, cardiovascular diseases, metabolic disorders, musculoskeletal diseases, and immunity related diseases.^1^^,^^2^^,^^3^

Aging is associated with a phenomenon termed ‘microvascular rarefaction’ where capillary density diminishes with age across various organs.^4^^,^^5^ Importantly, rarefaction precedes the appearance of cellular hallmarks of aging,^5^ which posits the possibility that vascular decline drives organismal decrease in tissue and cell functions and the occurrence of the age-related diseases. In line with this hypothesis, the prevention of vascular decline alleviates age-associated cognitive decline and insulin resistance and extends lifespan.^6^^,^^7^^,^^8^ Consistently, vascular repair or neovascularization is reduced with age in murine models of ischemic injury.^9^^,^^10^

Vascular maintenance and repair depend on the proliferative capacity of endothelial cells (ECs), the cells lining the inner side of vessels and forming the capillaries. Vascular repair following ischemic injury relies heavily on angiogenesis; the *de novo* capillary formation from preexisting vessels via ECs migrating toward a gradient of vascular endothelial growth factor (VEGF), supported by highly proliferating stalk EC forming the neovessel. In the context of muscle ischemia, muscle regeneration coincides with angiogenesis to restore oxygen, nutrient, and growth factor delivery to the ischemic tissue to promote myogenesis.^11^^,^^12^ ECs also influence macrophage differentiation and identity via angiocrine Notch signaling to promote arteriogenesis, i.e., the development of collateral vessels from pre-existing blood vessels, after hindlimb ischemia.^13^^,^^14^^,^^15^

Sprouting angiogenesis is in a large part fueled by metabolic reprogramming of EC and the upregulation of anaerobic glycolysis under hypoxic conditions.^16^^,^^17^ Furthermore, glucose-derived ATP and lactate directly regulates EC phenotypes (proliferative/quiescent)^16^^,^^18^ and proliferation.^19^

Nicotinamide adenine dinucleotide (NAD^+^) is an essential co-enzyme for cellular redox reactions, including glycolysis, the TCA cycle, and fatty acid oxidation.^20^^,^^21^ NAD^+^ levels decline during aging^20^^,^^22^^,^^23^^,^^24^^,^^25^ and supplying aged mice with the NAD^+^ precursors nicotinamide riboside (NR) and nicotinamide mononucleotide (NMN) improve health and longevity.^26^ NAD^+^ precursors also increased EC angiogenic capacity *in vitro,*^27^ and improves neovascularization following ischemic injury in elderly mice.^22^ However, the mechanisms underlying impaired NAD^+^ production and the exact effect of NAD^+^ precursors on EC metabolism remain unclear.

Here, we observed that while old ECs expressed elevated levels of pro-angiogenic genes at baseline, they are restrained by a reduced proliferation capacity stemming from impairments in NAD^+^-dependent metabolism. Our data demonstrates that supplementation with the NAD^+^ precursor NMN boosts angiogenic capacity in old mice and may offer an avenue to treat age-related decline in tissue perfusion and improve recovery after ischemia.

## Results

### Reduced EC proliferation capacity impairs post-HLI neovascularization response in aged mice

**Figure 1:**
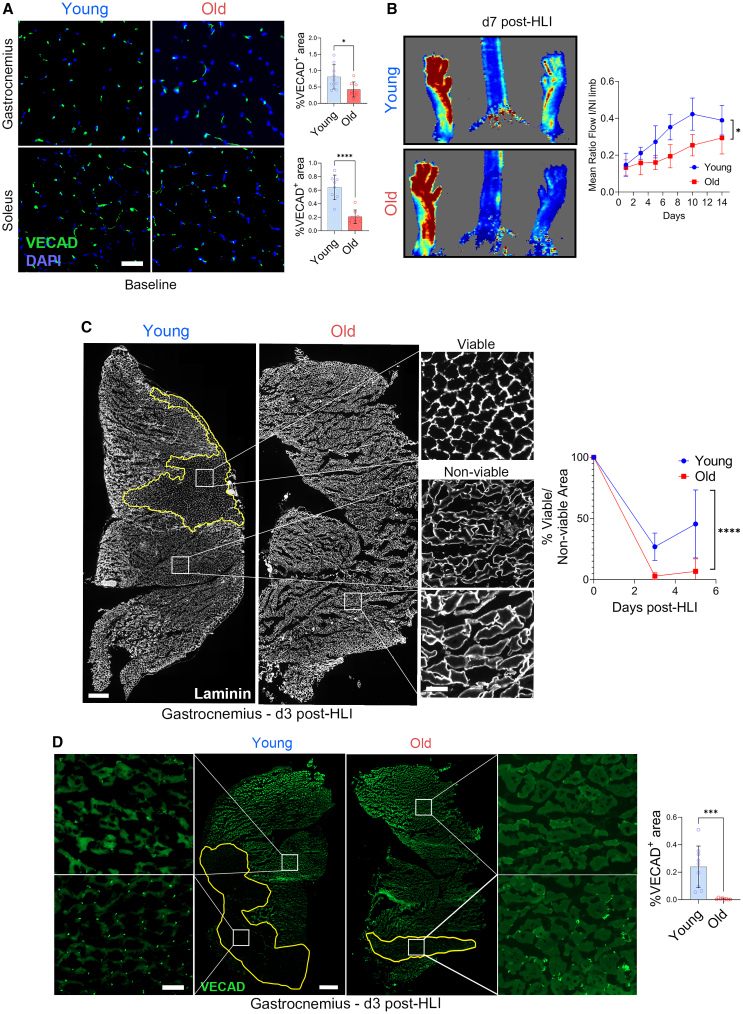
Aged mice undergo slower neovascularization and suffer more muscle damage post-hindlimb ischemia (A) Representative images (left) of VE-cad (green) and nuclei (blue) and quantification (right) of transverse sections of gastrocnemius and soleus muscle microvasculature in 4 (young) or 18 months (old) old male mice. n = 8–9 per group. Scale bar represents 50μm. ∗*p* ≤0 .05, ∗∗∗∗*p* <0 .0001 by unpaired bilateral Student’s *t* test. (B) Laser Doppler Perfusion Imaging (LDPI) post HLI as indicated. Data are mean ± S.D. of the ratio of ischemic over non-ischemic limbs. *n* = 8 per group. ∗*p* ≤ 0.05 as determined by unpaired bilateral Student’s *t* test of area under the curves between day 0 and 14. (C) Representative transverse sections (left) and quantification (bottom right panel) of laminin staining of gastrocnemius muscle at 3 days post-HLI. n = 4–8 per group. Scale bar represents 500μm (main) and 50μm (inset). ∗*p* ≤ 0.05 as determined by unpaired bilateral Student’s *t* test of area under the curves between day 0 and 5. (D) Ischemic gastrocnemius muscle microvasculature at 3 days post-HLI as depicted by representative images (left) and quantification (right) of transverse sections stained with VE-cad. Areas within bordered white line represent areas with well-ordered, viable vasculature. n = 8–9 per group. Scale bar represents 800μm (main) and 100μm (inset). Data are expressed as mean ± S.D. ∗*p* ≤0 .05, ∗∗*p* ≤0 .01, ∗∗∗*p* ≤0 .001 and ∗∗∗∗*p* ≤0 .0001 by unpaired bilateral Student’s *t* test.

When compared to young (3-month-old) mice, aged (18-month-old) mice displayed reduced capillary density of the gastrocnemius, soleus, and adductor muscles (Figures 1A and S1A). To explore the effects of aging on vascular functionality, we utilized the murine hindlimb ischemia (HLI) model, which involves the unilateral ligation of the femoral artery.^28^ When subjected to HLI, we observed an equivalent reduction in tissue perfusion in the ischemic hindlimb in both young and aged mice but a slower recovery of tissue perfusion in the aged mice (Figure 1B). At days 3 and 5 post-HLI, muscle damage was increased within the ischemic gastrocnemius muscle of aged mice (d3: 9.3-fold, d5: 6.7-fold larger; Figure 1C). Concurrently, VE-Cadherin (VE-Cad; EC marker) signal was almost undetectable at day 3 post-HLI on the ischemic gastrocnemius (Figure 1D) and soleus muscles (Figure S1B) of aged mice, while being clearly detectable in young mice, even within the damaged regions of the gastrocnemius and soleus muscles (Figure S1C). The VE-Cad positive signal remained lower in old gastrocnemius at day 21 post-HLI (Figure S1D).

**Figure 2:**
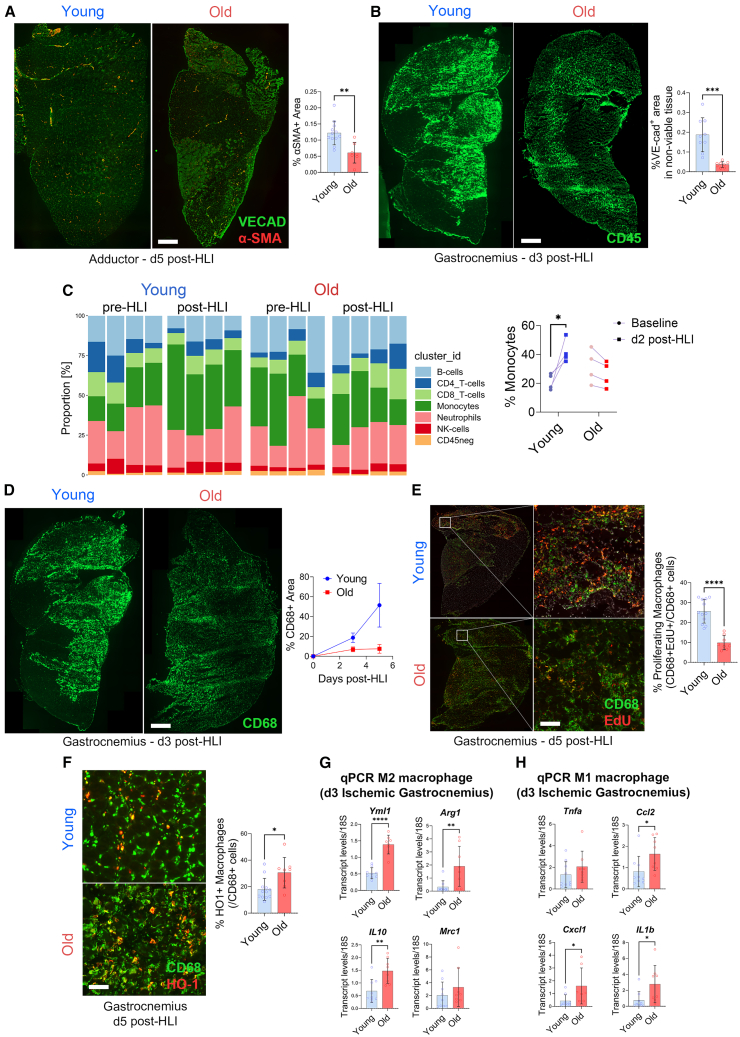
Arteriogenesis defect correlates with impairment in immune recruitment in aged mice muscle post-hindlimb ischemia (A) Representative images (left) and quantification (right) of ischemic adductor muscle stained with VE-cad and α-SMA at 5 days post-hindlimb ischemia in young and old WT male mice n = 8–12 per group. Scale bar represents 800μm. (B) CD45^+^ cell infiltration in gastrocnemius muscle at 3 days post-hindlimb ischemia. Representative images (left) and CD45^+^ area quantification (right). n = 8–9 per group. Scale bar represents 500μm. (C) Frequencies of B cells, T cells, monocytes, neutrophils, NK and CD45^−^ cells at baseline and at 2 days post-HLI in young and old male WT mice. Percentage of monocyte of total myeloid cells at baseline and at 2 days post-HLI (right). ∗*p* ≤ 0.05 by adjusted paired bilateral Student’s *t* test. (D) Representative images (left) and quantification (right) of CD68^+^ infiltration in gastrocnemius muscle at 3 or 5 days post-hindlimb ischemia. n = 8–10 per group. Scale bar represents 500μm. (E) Proliferating macrophages in gastrocnemius muscle at 5 days post-hindlimb ischemia as depicted by representative images (left) and quantification (right) of transverse sections stained with CD68 and EdU. n = 8–10 per group. Scale bar represents 100μm. (F) M2-like macrophages in gastrocnemius muscle at 5 days post-hindlimb ischemia as depicted by representative images (left) and quantification (right) of transverse sections stained with CD68 and EdU. n = 8–10 per group. Scale bar represents 100μm. (G and H) Quantitative real-time PCR gene expression analysis of (G) M2 macrophage (H) M1 macrophage markers in ischemic gastrocnemius muscle at 3 days post-hindlimb ischemia. All genes were normalized to 18S expression. n = 8–9 per group. (A and B, D–H). Data are expressed as mean ± S.D. ∗*p* ≤ 0.05, ∗∗*p* ≤ 0.01, ∗∗∗*p* ≤ 001 and ∗∗∗∗*p* ≤ 0.0001 by unpaired bilateral Student’s *t* test.

The signal for smooth muscle alpha-actin (α-SMA, an arterial marker) was 2.6-fold lower in the ischemic adductor muscle of the aged mice at day 5 post-HLI (Figure 2A), suggesting decreased arteriogenesis. Of note, the α-SMA staining and arteriole number in the adductor muscle was similar between young and aged mice in non-ischemic adductors (Figure S1E). Arteriogenesis relies on M2 macrophages to support vascular remodeling.^14^^,^^15^ At day 3 post-HLI, infiltration of CD45^+^ myeloid cells (Figure 2B) was reduced 2.5-fold in the ischemic gastrocnemius muscle of aged mice. To gain further insight, we analyzed peripheral blood cells with mass cytometry (CyTOF) and performed an unsupervised clustering algorithm to distil 20 different clusters, which could be annotated to 7 cell types (Figure S2). At baseline, we observed no age-related differences in any of the annotated cell subsets. However, at day 2 post-HLI, the percentage of circulating monocytes was increased in young mice, but not in old mice. No significant differences were observed in other cell subsets post-HLI (Figure 2C). Consistently, CD68^+^ macrophages infiltration in the gastrocnemius muscle was reduced 2.7-fold at day 3 and 6.9-fold at day 5 post-HLI in aged mice (Figure 2D). Moreover, macrophage proliferation was reduced 2.6-fold in aged mice at day 5 post-HLI (Figure 2E). CD68^+^ HO1^+^ M2-like macrophages were increased 1.7-fold in the ischemic calf muscle of aged mice at day 5 post-HLI (Figure 2F), suggesting a shift toward pro-repair M2 macrophage in the old mice. However, qPCR analyses revealed significant increases in the expression of both M2-like macrophages markers (Arg1, Yml1, and IL10; Figure 2G) and M1-like pro-inflammatory macrophages markers (Ccl2, IL1b, Cxcl1; Figure 2H). Overall, aging was associated with a mild impairment in arteriogenesis and macrophage infiltration despite a pro-inflammatory environment. However, tissue-infiltrating macrophages may be shifted toward a pro-repair phenotype in aged tissues, which usually supports neovascularization. Thus, we then focused on sprouting angiogenesis.

**Figure 3:**
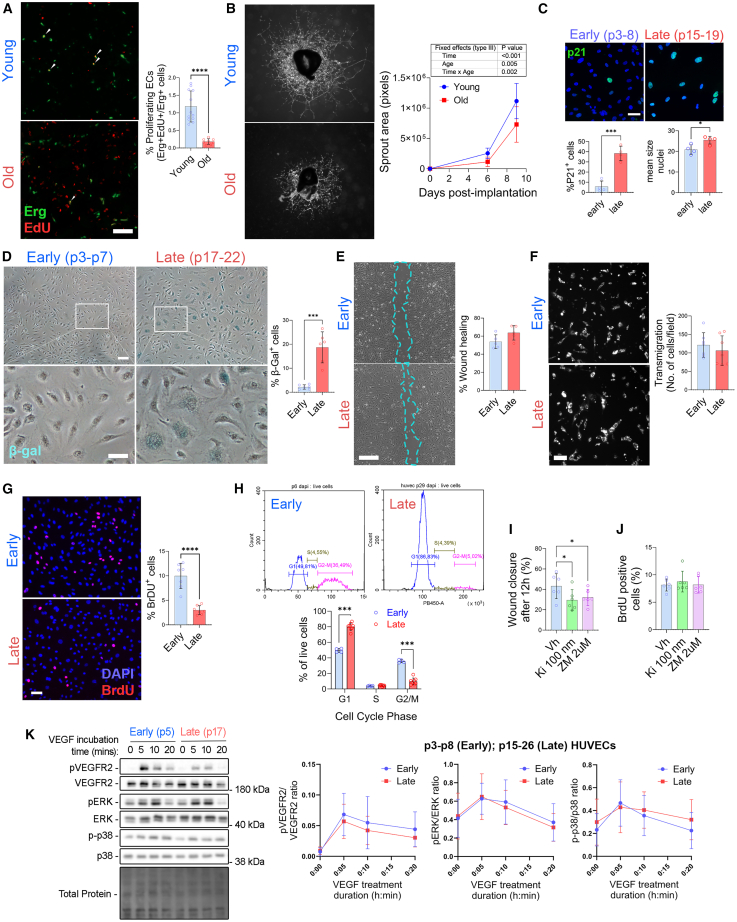
Impairment of EC proliferation results in neovascularization defect in old mice (A) Proliferating ECs in gastrocnemius muscle at 5 days post-hindlimb ischemia. Representative images (left) and quantification (right) of gastrocnemius stained with Erg and EdU. n = 8–10 per group. Scale bar represents 100μm. (B) Average area of microvessel sprouting from aortic ring explants from young or old mice incubated in full EGM2 media. Images at day 9 post-implantation (left) and quantification (right). Data are mean ± S.D. of n = 8–10 per group. ∗∗*p* ≤ 0.01 by Mixed Effect analysis (REML). Scale bar represents 100μm. (C) P21^CIP^ immunocytochemistry (green) and nuclei (DAPI; blue) in early and late passage HUVECs. Scale bar represents 50μm. Percentage of P21^CIP^ positive cells and mean nuclei size in four independent experiments. (D) β-galactosidase^+^ (β-gal; senescent) in early and late passage HUVECs. *n* = 6 per group. Scale bar represents 50μm (main) and 20μm (inset). (E) Representative image (left, 10× magnification) and quantification (right) of early and late passage HUVECs in a wound healing scratch assay. *n* = 5 per group. Scale bar represents 15μm. (F) Early- and late-passage HUVEC transmigration through the polycarbonate membrane of a Boyden chamber from unsupplemented EBM-2 medium toward supplemented EGM-2 medium. Scale bar represents 50 μm. (G) BrdU and DAPI positive nuclei in early and late passage HUVECs. Representative images (left) and quantification (right). *n* = 7 per group. Scale bar represents 20μm. (H) DNA content assessment by flow cytometry in early and late passage HUVECs. Representative flow cytometry plot of DAPI expression against cell count (left) and quantification of the proportion of live cells within G1, S and G2/M phases of the cell cycle (right). n = 4–7 per group. (I) Migration across a scratch and (J) BrdU incorporation of early passage HUVECs treated with the VEGF inhibitors KI8751 (100 nM) and ZM323881 (2 μM). *n* = 6 per group. (K) Western blot on lysates of early and late passage HUVECs treated with 20nM VEGF-A_165_ for 0, 5, 10, and 20 min. P-VEGFR2, P-ERK and P-p38 levels normalized with total VEGF, ERK and p38 levels respectively. (A–F) Data are shown as mean ± S.D. ∗*p* ≤ 0.05, ∗∗*p* ≤ 0.01, ∗∗∗*p* ≤ 0.001 and ∗∗∗∗*p* ≤ 0.0001 by bilateral unpaired Student’s *t* tests (A, C–G) or matched Mixed-effects model (REML) (B, H–K) followed by Dunnett’s multiple comparisons tests (H–I).

We observed a 6.4-fold reduction in the proportion of proliferating endothelial cells (EdU^+^Erg^+^) in the calf muscles of aged mice at day 5 post-HLI (Figure 3A). Moreover, sprouting angiogenesis from aortic explants was reduced 2-fold in aged compared to young mice (Figure 3B). To examine further the phenotype of aged EC, we utilized an *in vitro* model of EC aging comparing early (1–10) and late passages (>15) of primary human umbilical vein endothelial cells (HUVECs).^29^^,^^30^^,^^31^ The late passage HUVECs displayed feature of replicative senescence with nuclear expression of the senescence marker cyclin-dependent kinase inhibitor p21^WAF1/Cip1^ and accumulation of larger cells with enlarged nuclei (Figures 3C and S3A) expressing β-galactosidase (Figure 3D) and overexpressing p16^INK4A^ (Figure S3A). While migratory capacity was maintained (Figures 3E and 3F), proliferation was reduced by 3.3-fold reduction in late passage HUVECs (Figure 3G). Moreover, late passage HUVEC accumulated in the G1 phase and displayed increased level of the G1 phase marker Cyclin D1 (Figures 3H and S3A).

Inhibition of VEGF signaling using two VEGFR inhibitors KI8751 and ZM323881 impacted cell migration (Figure 3I), but not proliferation in early passage HUVECs (Figure 3J). In line with this observation, there was no difference between early and late HUVECs in human VEGF_165_-induced phosphorylation of vascular endothelial growth factor receptor 2 (VEGFR2), extracellular signal-regulated kinase (ERK) and p38 (Figure 3K). Thus, the defective cell proliferation in old ECs was not due to impaired VEGF-A response or cell-cycle arrest.

### Aged ECs display altered gene expression in angiogenic and metabolic pathways

**Figure 4:**
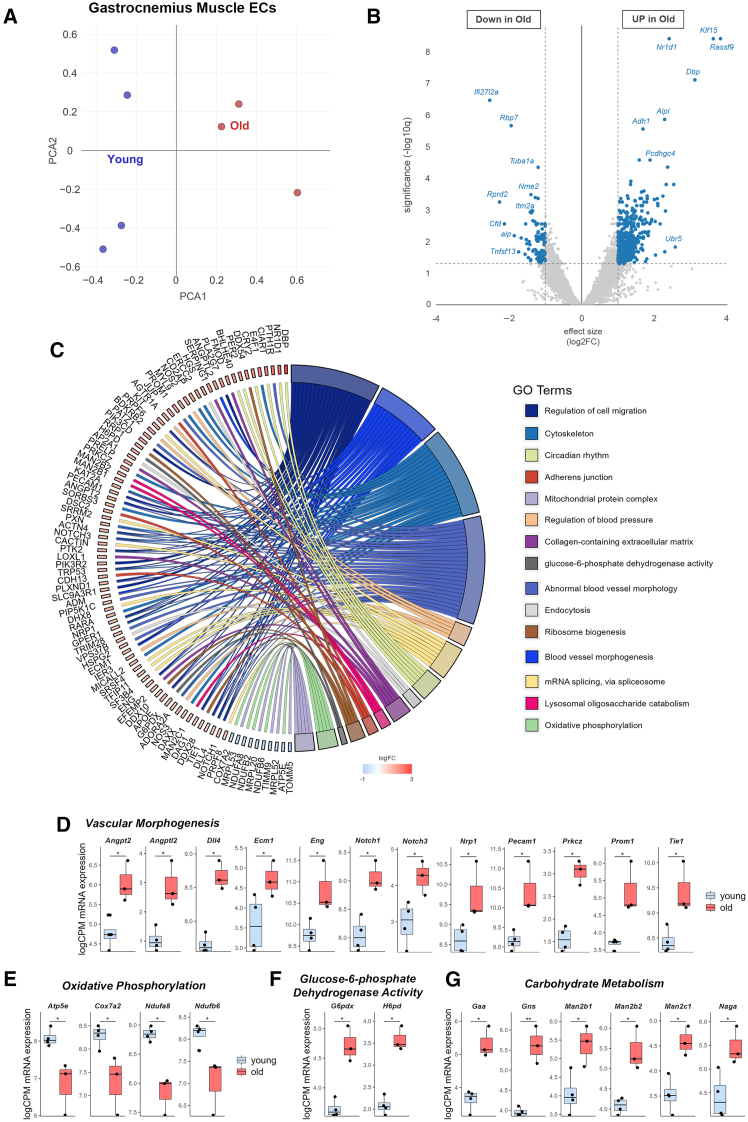
Old ECs exhibit an altered gene expression profile (A) PCA plot visualizing the degree of similarity in gene expression profiles of individual samples of young (blue) and old (red) ECs. n = 3–4 per group. (B) Volcano plot representing the identification of 500 DEG (aggregated q value of 0.05, and fold change = 2) with age in skeletal muscle ECs. n = 3–4 per group. (C) Chord diagram representing the connections between DEGs and functional pathways, differentiated by color (right). n = 3–4 per group. (D–G) mRNA expression of genes associated with vascular morphogenesis (D), oxidative phosphorylation (E), glucose-6-phosphate dehydrogenase activity (F), carbohydrate metabolism (G), expressed as logarithm of counts per million reads (logCPM). Statistical significance determined through false discovery rate calculated by the method of Benjamini, Hochberg, and Yekutieli; ∗q <0 .05, ∗∗q < 0.01.

To understand the age-related impaired EC proliferation, we undertook an unbiased comparison of the gene expression profiles of ECs from the gastrocnemius muscle harvested from young and old mice. Unsupervised clustering demonstrated a clear divergence in gene expression phenotype between young and old ECs (Figure 4A). Setting a significant threshold at q < 0.05 with a fold change >2, we identified 500 differentially expressed genes (DEGs; 406 upregulated, 94 downregulated; Figure 4B; Table S1). Gene network analysis of DEGs revealed 12 clusters, 10 upregulated and 2 downregulated (Figure 4C).

Interestingly, we observed an upregulation of a large gene cluster associated with pro-angiogenic vascular morphogenesis in aged ECs (Figure 4D), most of which is represented within the 100 most significant genes within the previously described analysis (Figure S4A). This cluster contains Notch1 and Dll4, genes central in tip-stalk cell speciation during angiogenesis,^32^^,^^33^^,^^34^ Nrp1, a VEGF receptor vital for EC migration toward a VEGF gradient,^35^ the growth factor Angpt2, which enhances tip cell migration and vascular sprouting,^36^ and Tie1, which is required for full activation of Tie2 by Angpt1/Angpt2.^37^^,^^38^ Aged muscle ECs also exhibited alterations in metabolic gene expression levels. Genes involved in oxidative phosphorylation were downregulated (Figure 4E), while genes associated with the pentose phosphate pathway (G6pdx and H6pd) and carbohydrate metabolism (e.g., Gaa, Gns) were upregulated (Figures 4F and 4G). These metabolic adaptations are reminiscent of EC metabolic reprogramming in the context of angiogenesis.^39^^,^^40^^,^^41^^,^^42^^,^^43^ Although metabolic gene expression is not equivalent to metabolic fluxes, there is some evidence that metabolic gene expression can predict metabolic activity.^44^^,^^45^ Computational prediction of metabolic pathway variation was performed as previously described.^45^ This analysis suggested that gene expression within the glycolysis and TCA cycle pathways were upregulated, while oxidative phosphorylation was downregulated (Figure S4B).

### Aged endothelial cells exhibit significant metabolic alterations, including distinct glycolytic and TCA cycle dynamics

**Figure 5:**
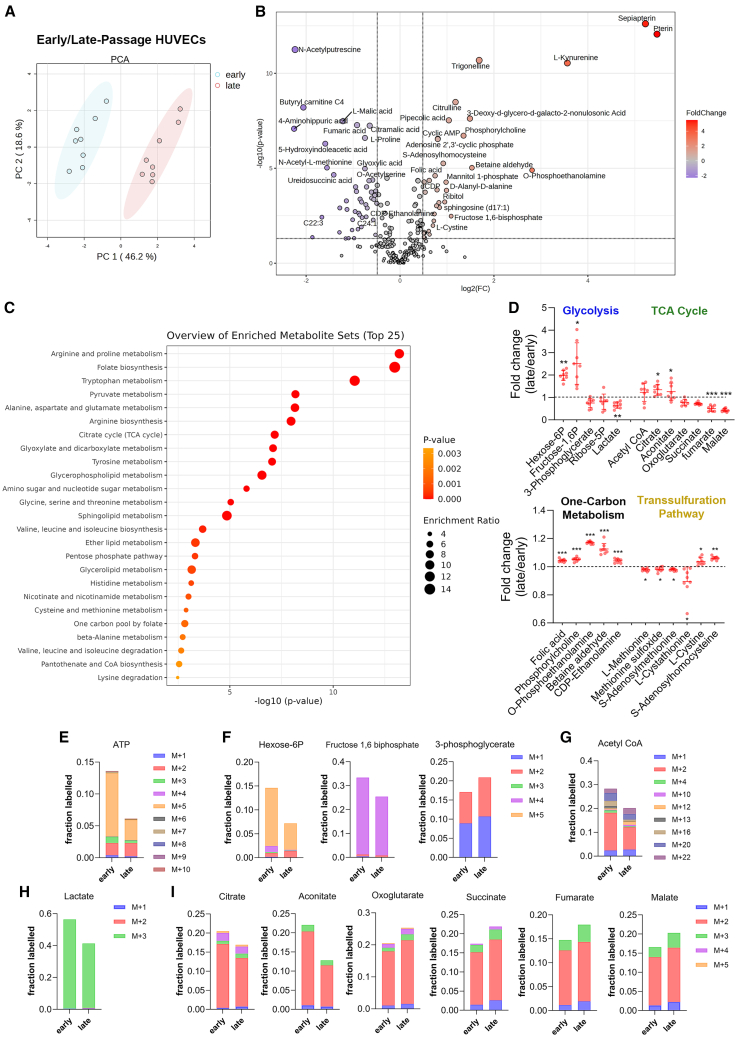
Late passage HUVECs exhibit vast changes in metabolism (A) Principal component analysis clustering of polar metabolites from early- and late-passage HUVECs. (B) Volcano plot representing the identification of metabolites that are differentially expressed with age in early- and late-passage HUVECs with a q value > 0.05 and a fold change (FC) > 1.4. (C) Pathway enrichment analysis comparing early- versus late-passage HUVECs. (D) Relative abundance of individual metabolites expressed as fold change in one-carbon metabolism, transsulfuration, glycolysis and TCA cycle pathways, with mean ± S.D. Statistical significance determined through false discovery rate calculated by the method of Benjamini, Hochberg, and Yekutieli; ∗q <0 .05; ∗∗q <0 .01; ∗∗∗q <0 .001. *n* = 8 per group. (E–I) Glucose-derived ^13^C enrichment into (E) ATP, (F) intermediates of the glycolysis pathway, (G) lactate, (H) acetyl CoA, (I) and intermediates of TCA cycle.

Recognizing that metabolic gene expression does not necessarily reflect actual metabolic fluxes, we conducted targeted metabolomic analyses in early and late passage HUVECs to explore this relationship in greater depth. Principal component analysis (Figure 5A), volcano plot (Figure 5B), and heatmap representation (Figure S5A) revealed a clear variance between the two groups with 48 significantly down-regulated, and 37 up-regulated metabolites (Figure 5B; Table S2). Pathway enrichment analysis further highlighted alterations in amino acid metabolism, especially in arginine, aspartate, and glutamate metabolism (Figure 5C; Table S3).

Overall, amino acids were depleted in late passage HUVECs, with significant downregulation of aspartate, valine, proline, phenylalanine and threonine, and a tendency toward decreased levels of glutamate, glutamine, alanine, and leucine (Figure S5B). A more in-depth examination of the metabolites annotated in the pathway analysis revealed that the enrichment of these pathways was predominantly driven by significant alterations in TCA cycle intermediates. In the TCA cycle, citrate and aconitate were increased, while the later intermediates fumarate and malate were depleted (Figure 5C). The related urea pathway also showed significant changes in late passage HUVEC (Figure S5C). In the glycolysis pathway, glucose-6-phosphate and fructose-1,6-biphosphate were increased, while the rest of the pathway tended to be decreased in late passage HUVECs (Figure 5D). We also observed alterations in the one-carbon metabolism with large accumulation of folate, betaine, phosphorylcholine and phosphoethanolamine, and depletion of methionine, S-adenosyl methionine and cystathionine, and accumulation of homocysteine (Figure 5D). The pentose phosphate pathway (PPP) intermediate ribose-5-phosphate and the serine biosynthesis pathway (Figure S5D) were unaffected.

**Figure 6:**
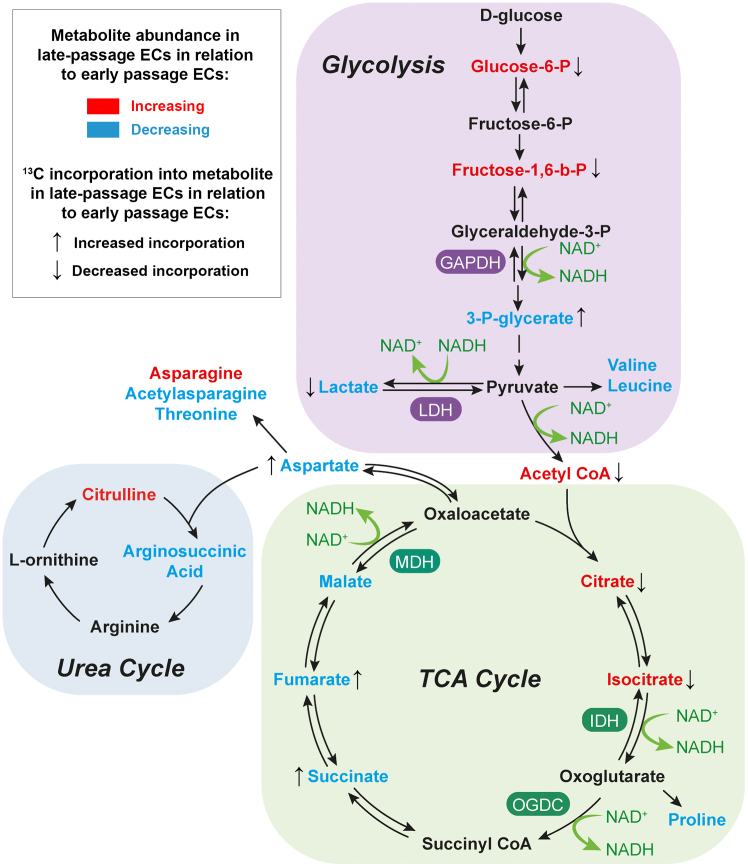
Schematic diagram summarizing the glycolytic and TCA cycle intermediates in late-passage HUVECs in relation to early-passage HUVECs (*n* = 8 per group) Differential metabolite abundances are described as increased (red) and decreased (blue) in late-passage HUVECs in relation to early-passage HUVECs. ^13^C incorporation are described as increased (↑) and decreased (↓) in late-passage HUVECs in relation to early-passage HUVECs.

To gain more insight into the metabolic changes within late passage HUVECs, these cells were cultured for 3 h in presence of uniformly labeled ^13^C glucose. First, ^13^C incorporation into ATP was severely reduced in late passage HUVECs (Figure 5E). ^13^C enrichment in glucose-6-phosphate and fructose-1,6-biphosphate, but not 3-phosphoglycerate was also reduced in late passage HUVECs (Figure 5F). ^13^C incorporation in acetyl CoA and lactate was also reduced (Figures 5G and 5H), indicating lower glycolytic activity in late passage HUVECs. In addition, ^13^C incorporation in the early TCA cycle intermediates acetyl CoA, citrate and aconitate was reduced, with a shift toward lower magnitude incorporation, suggesting fewer cycles. However, ^13^C incorporation in the depleted metabolites α-ketoglutarate, succinate, fumarate, and malate was increased, suggesting rewired metabolism toward reductive carboxylation because of defective TCA cycle. In line with our metabolomics findings, we observe no significant changes in ^13^C incorporation within the intermediates of the PPP and serine biosynthesis pathway (Figures S5E and S5F). Interestingly, we observed a reduction in aspartate levels and increased levels of glucose-derived ^13^C incorporation in aspartate (Figure S5G). Changes in the abundance and ^13^C incorporation into metabolic intermediates in late passage HUVECs are summarized in a schematic diagram (Figure 6).

### Age-associated NAD^+^ deficiency in ECs contributes to sub-optimal neovascularization capacity

**Figure 7:**
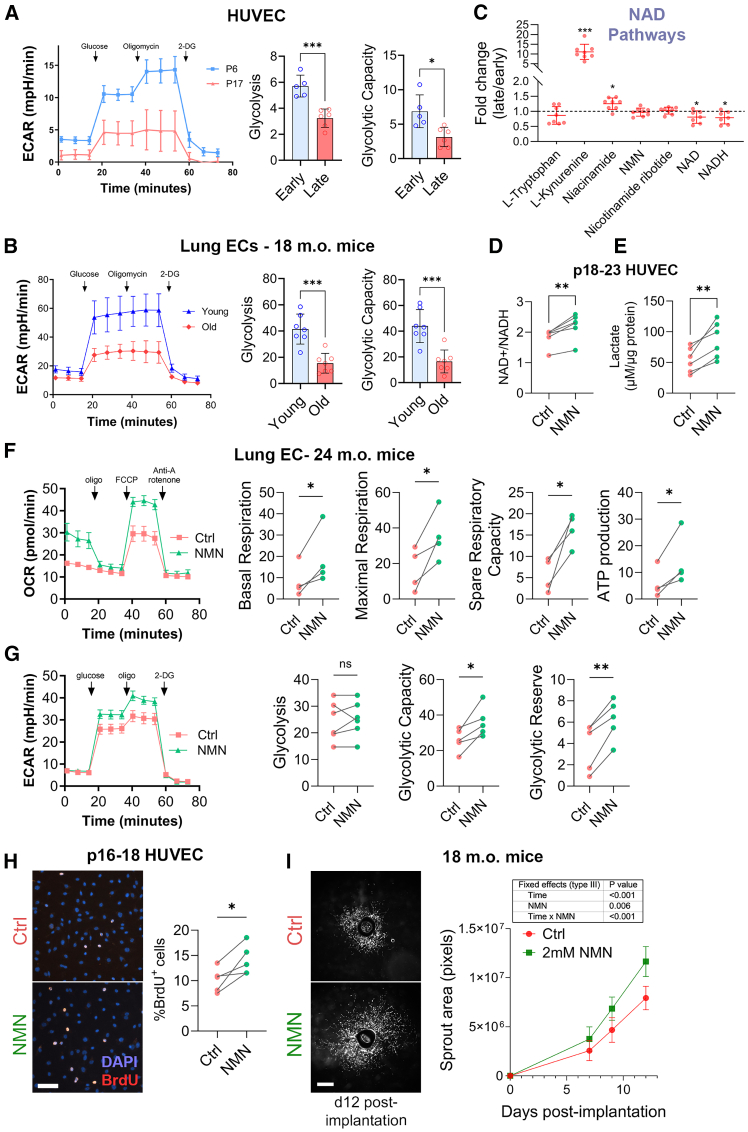
Glycolytic capacity is impaired in late-passage HUVECs and aged lung ECs due to diminished NAD⁺ abundance (A and B) Glycolysis stress test in (A) early and late passage HUVECs, and (B) ECs harvested from 3- or 18-month-old mice. Glycolysis is measured by extracellular acidification rate (ECAR). ∗*p* <0 .05, ∗∗∗*p* <0 .001 by unpaired bilateral Student’s *t* tests. Representative mean ± S.D. traces and quantification from 4 to 5 animals. (C) Relative abundance of individual metabolites expressed as fold change in the kynurenine pathway, with mean ± S.D. Statistical significance determined through false discovery rate calculated by the method of Benjamini, Hochberg, and Yekutieli. ∗q <0 .05; ∗∗∗q <0 .001. *n* = 8 per group. (D) Quantification of NAD^+^ over NADH concentration in late passage HUVECs exposed or not (Ctrl) to 2mM NMN for 24h (5 independent experiments). ∗*p* <0 .05 by paired bilateral Student’s *t* tests. (E) Lactate concentration in the supernatant of late passage HUVECs exposed or not (Ctrl) to 2mM NMN for 24h (5 independent experiments). ∗*p* <0 .05 by paired bilateral Student’s *t* tests. (F and G) Seahorse Mitochondria (F) or Glycolysis (G) stress test in lung ECs harvested from 24-month-old male mice treated or not (Ctrl) for 4 h with 2mM NMN. ∗*p* <0 .05 by paired bilateral Student’s *t* tests from 5 independent experiments. (H) Proliferating BrdU^+^ over DAPI nuclei. Representative images (left) and quantification (right) of late passage HUVECs treated or not (Ctrl) with 0.5 mM NMN. Scale bar represents 50μm. ∗*p* <0 .05 by paired bilateral Student’s *t* test on 4 independent experiments. (I) Average area of microvessel sprouting from aortic ring explants from old mice incubated in EGM2 media (Ctrl) or EGM2 media supplemented with 2mM NMN as represented by images at day 12 post-implantation (left) and quantification (right). Scale bar represents 200μm. *p* values as indicated as determined by matched (repeated measures) Mixed-effects model (REML).

Functional analysis by Seahorse confirmed that glycolytic capacity was reduced both in late passage HUVECs and in lung ECs harvested from aged mice (Figures 7A and 7B). Lung EC were used as a better model of old EC because muscle EC metabolism could be influenced by lower activity levels in old animals, leading to a bias in the profile of muscle EC as muscle activity deeply influence the EC phenotype and gene expression profile.^46^^,^^47^ Our data suggested blockage at the steps catalyzed by glyceraldehyde-3-phosphate dehydrogenase (GAPDH) in glycolysis and isocitrate dehydrogenase (IDH) in the TCA cycle (as summarized in Figure 6). Interestingly, both rate-limiting enzymes are NAD^+^-dependant,^48^ suggesting a link with NAD^+^ availability. In line with this hypothesis, L-kynurenine accumulated 10-fold in late passage HUVECs (Figure 7C), suggesting compromised *de novo* NAD^+^ synthesis via the kynurenine metabolic pathway.^49^^,^^50^ The NAD^+^ salvage pathway may also be impacted with accumulation of NAD^+^ precursor niacinamide, while NMN and ribotide were unaffected. Finally, both NAD^+^ and NADH levels were significantly lower in late passage HUVECs (Figure 7C), supporting our hypothesis that the impaired GAPDH and IDH reactions may be caused by NAD^+^ depletion.

To test this hypothesis, late passage HUVECs were supplemented with the NAD precursor NMN to boost NAD^+^ production.^51^^,^^52^^,^^53^ A 24 h 1mM treatment with NMN increased the NAD+/NADH ratio (Figure 7D), and the accumulation of extracellular lactate in late passage HUVECs (Figure 7E), indicative of an increase in glycolytic activity. A 1mM NMN treatment for 4 h increased by 2 to 3-fold oxidative phosphorylation in lung ECs harvested from 24-month-old male mice (Figure 7F). 1mM NMN treatment for 4 h also boosted glycolytic capacity and reserve, but not basal glycolysis in lung ECs harvested from 24-month-old male mice (Figure 7G). Late passage HUVECs treated with 0.5 mM NMN exhibited a 1.3-fold increase in cellular proliferation (Figure 7H). Furthermore, 2 mM NMN led to a 1.4-fold increase in sprouting angiogenesis in aortic explants harvested from 24-month-old male mice (Figure 7I).

### Beneficial effects of NMN supplementation requires the contribution of glycolysis-derived pyruvate to feed the mitochondrial TCA cycle

**Figure 8:**
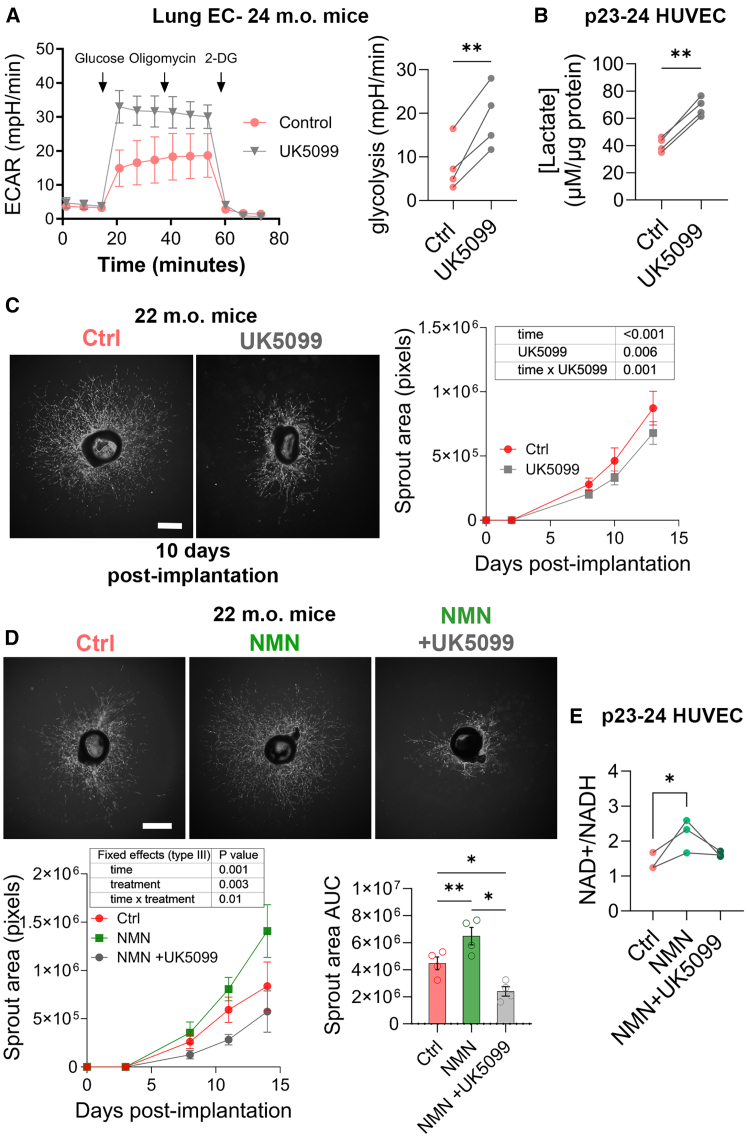
Inhibition of pyruvate transport into the mitochondria abrogates the beneficial effects of NMN supplementation (A) Glycolysis stress test in ECs harvested from 24-month-old male mice pre-treated or not (Ctrl) with 1μM UK5099 for 4h. Glycolysis is measured by extracellular acidification rate (ECAR). Traces are representative mean ± SD of 4 animals. ∗∗*p* <0 .01 by paired bilateral Student’s *t* tests. (B) Lactate concentration in the supernatant of late passage (p22 to 24) HUVECs exposed or not (Ctrl) to 1 μM UK5099 for 24h (4 independent experiments). ∗*p* <0 .05 by paired bilateral Student’s *t* tests. (C and D) Average area of microvessel sprouting from aortic ring explants from old male mice incubated in full EGM2 media supplemented with 2mM NMN and/or 1μM UK5099. Representative images at day 10–11 post-implantation (left) and quantification (right). Data are mean ± S.D. of *n* = 4 animals. Scale bar represents 200μm. ∗*p* ≤ 0.05, ∗∗*p* ≤ 0.01. *p* values were determined by matched (repeated measures) Mixed-effects model (REML) followed by Tukey’s multiple comparisons. (E) Quantification of NAD^+^ over NADH concentrations in HUVECs treated for 24h with 2mM NMN and 1μM UK5099 (*n* = 3). *p* <0 .05 by one-way ANOVA followed by Tukey’s multiple comparisons.

Sprouting angiogenesis is fueled by upregulation of anaerobic glycolysis,^16^^,^^17^ and glucose-derived ATP and lactate directly regulates EC proliferation.^19^ To promote lactate production cells were treated with a selective inhibitor for the mitochondrial pyruvate carrier 1 (MPC1), UK5099, which blocks pyruvate entry into the TCA cycle, thus promoting the use of pyruvate for lactate production.^54^ UK5099 strongly increased lactate as assessed by seahorse (Figure 8A) or by measuring accumulation of lactate in the supernatant of late passage HUVECs (Figure 8B). However, 1μM UK5099 reduced sprouting angiogenesis from aortic explants harvested from 22-month-old male mice (Figure 8C) and completely abolished the benefits of NMN on sprouting (Figure 8D). Of note, UK5099 also completely abolished the NMN-induced increase in NAD^+^ levels (Figure 8E).

## Discussion

In this study, we observed an age-related decrease in tissue capillary density as well as an impaired neovascularization response to ischemia and an impaired angiogenic potential in sedentary old male mice. Defective angiogenic potential was mainly caused by ECs failure to proliferate, secondary to metabolic dysfunction and ATP depletion. Finally, restoring NAD^+^ levels partially rescued old EC proliferative capacity, and might serve as future therapeutics.

Neovascularization in the HLI model requires arteriogenesis and M2-like macrophages to support vascular remodeling.^13^^,^^55^^,^^56^ Here, macrophage mobilization and infiltration following HLI were impaired in old compared to young mice. However, these macrophages seemed shifted toward a pro-repair M2 phenotype in old mice, suggesting a tissue macrophage-specific compensatory mechanism to support arteriogenesis. Further studies are required to better understand how the age-related pro-inflammatory microenvironment exactly contribute to the impaired neovascularization observed in aging. Neovascularization following ischemic injury also relies on sprouting angiogenesis.^57^^,^^58^ Here, we observed a reduction in the proliferative capacity of aged ECs in the ischemic muscle post-HLI, along with diminished aortic sprouting capability *ex vivo*. To understand the differences between young and old EC that might explain impaired sprouting angiogenesis, we compared the RNA signature of purified muscle ECs from young and old male sedentary mice. This revealed changes reminiscent of EC reprogramming in the context of angiogenesis,^39^^,^^40^^,^^42^^,^^43^^,^^59^^,^^60^ suggesting that while EC do not proliferate, they sense their environment and respond to the need for vascular repair. In line with this hypothesis, late passage HUVECs retain their abilities to migrate and respond to VEGF. Of note, we observed that VEGF signaling impacts early passage HUVEC migration but not proliferation. Overall, our results suggest that the age-related impairment in angiogenic potential stems from impaired EC proliferation, but not VEGF-mediated EC migration. It was previously showed that early VEGF overexpression extends lifespan.^7^ However, more studies are necessary to validate whether VEGF augmentation in old age can rejuvenate vascular function as our data point out that it may not be sufficient to restore impaired EC proliferation.

Our transcriptomics signature also suggests a reduction of oxidative phosphorylation and upregulation of glycolysis *in vivo* in old muscle EC, as previously described during angiogenesis.^61^^,^^62^ However, despite these changes, glycolytic capacity was decreased in aged ECs and late-passage HUVEC. In EC, glycolysis provides over 85% of ATP even in oxygen-replete conditions.^16^ Glucose-derived ATP and lactate also directly regulates EC phenotypes (proliferative/quiescent)^16^^,^^18^ and proliferation.^19^ Our metabolomics experiments further revealed that glucose-derived ATP and lactate production were reduced in late-passage HUVEC, which likely contributes to the defect in the energy-intensive process of cellular proliferation. In addition, we also observed changes in the TCA cycle. Of note, the fact that malate and fumarate are depleted in late-passage HUVEC is consistent with several human studies showing that TCA intermediates are lower in the elderly.^63^ It also confirms previous *in vitro* findings that replicative senescence in HUVECs is characterized by a substantial loss of cellular energy currency owing to the reduction of both glycolysis and mitochondrial respiration.^64^ Our data suggests that impaired glycolysis and TCA cycle may stem from compromised reactions of NAD^+^-dependent enzymes, including glyceraldehyde-3-phosphate dehydrogenase (GAPDH) and isocitrate dehydrogenase (IDH) enzymes, rate limiting enzymes in glycolysis and the TCA cycle, respectively.^65^^,^^66^ Of note, dysregulation of NAD^+^ metabolism is a well-known feature of aging^20^^,^^22^^,^^23^^,^^24^^,^^25^ and we confirmed using late-passage HUVEC that NAD^+^ is depleted in old EC.^22^ We further observed that the NAD^+^ precursor NMN improves late passage HUVEC proliferation and sprouting angiogenesis in old mice. Importantly, our findings concur with a previous study describing that NAD^+^ depletion impairs ECs proliferation, but not migration.^67^ To evaluate the role of glycolysis and lactate production in angiogenesis, we assessed the impact of UK5099, which blocks pyruvate entry into the TCA cycle, thus promoting the use of pyruvate for lactate production. UK5099 has been extensively used in cancer research to promote the Warburg effect and is known to favor tumor survival.^54^ Here, the glycolysis booster UK5099 inhibited sprouting angiogenesis and abolished the effect of NMN, underscoring the importance of the TCA cycle in EC proliferation and TCA-driven OXPHOS in NAD^+^ production. These findings are in line with previous studies showing that the electron transport chain plays a major role in proliferation via an impact on aspartate synthesis for purine formation.^68^^,^^69^^,^^70^ We also observed a reduction in aspartate abundance alongside an accumulation of folate cycle intermediates, indicative of impaired folate cycling in late passage human ECs. Amino acid depletion was actually a major feature of late-passage HUVECs, and aligns with a similar study showing that replicative senescence is associated with reduced amino acid, especially valine, proline and aspartate,^64^ as observed here. Furthermore, NMN supplementation mainly promoted oxidative phosphorylation in late passage human ECs, suggesting that impaired TCA cycle rather than glycolytic capacity if the main limitation for old EC proliferation.

More broadly, our *in vitro* data may reveal impaired one-carbon metabolism (OCM) network with large accumulation of folate, betaine, phosphorylcholine, phosphoethanolamine and homocysteine. The OCM regroups the folate cycle and methionine cycle and regulate purine and thymidine synthesis, amino acid homeostasis, and epigenetic mechanisms, thereby regulating cellular survival, growth, proliferation, stress response, and aging.^71^^,^^72^ Interestingly, some of the age-associated changes in OCM intermediates we observed have also been observed in human studies,^63^ which posits the intriguing idea that aged ECs contribute to the circulating metabolite signature of the elderly.

The decline of NAD⁺ levels in aged ECs is a multifaceted phenomenon with potential origins spanning various cellular processes. Our late-passage HUVEC data suggest an impairment in the kynurenine metabolic pathway, the primary pathway for *de novo* NAD^+^ synthesis, as well as the NAD^+^ salvage pathway. Furthermore, previous studies have established that the H_2_S signaling network regulates NAD^+^ levels.^22^ Interestingly, our findings also underscore changes in the transsulfuration pathway, the main pathway involved in H_2_S production. These findings suggest that H_2_S production may be compromised, which aligns with previous studies describing impaired H_2_S production in aging.^22^^,^^73^

Replenishing NAD^+^ levels has been shown to reverse some of these changes and restores proliferation capacity.^70^^,^^74^ Interestingly, it was shown that NMN treatment improves blood flow post HLI and exercise capacity in elderly male mice.^22^ We recently demonstrated that NMN treatment does not promote EC proliferation in young ECs.^28^ Similarly, NMN supplementation has no effect on the capillary density of sedentary young to middle-aged mice,^22^ which support the safety of NAD^+^ precursor supplementation, which merely provides a necessary depleted cofactor, but does not alter young EC function. Although previous studies have identified SIRT1 as a key mediator of the benefits associated with NAD^+^ precursor supplementation,^22^ our findings suggest that the benefits may also be attributed to an enhanced availability of glycolysis-derived pyruvate. Thus, our data suggest that glycolysis-derived pyruvate serves as a critical substrate for the mitochondrial TCA cycle to support biomass production and NAD^+^ regeneration essential for EC proliferation.

Accumulation of senescent ECs drives age-associated pathologies such as diabetes, hypertension, and atherosclerosis.^75^ However, the impact of EC senescence in natural aging remains unclear, with tissue variable senescent ECs accumulation, and conflicting evidence showing that removal of senescent cells could have either positive or negative impacts on tissue function.^76^^,^^77^ While we did not observe an overexpression of senescence markers in old EC harvested from 18-month-old mice (∼50–60-year-old human), previous studies have described overexpression of senescence markers in ECs harvested from healthy, >24-month-old mice^76^^,^^77^^,^^78^(∼70 year old human) and >75-year-old humans in different vascular beds.^79^^,^^80^ Thus, our data suggest that the NAD^+^-dependent defect in EC proliferation precedes senescence, and further studies are required to dissect its role in senescence induction and its age-associated pathologies.

### Conclusions

In this study, we combined *in vivo* and *in vitro* experiments, incorporating RNA sequencing, metabolomics, and functional assays of metabolism, proliferation, and migration. We propose that the age-related decline in angiogenesis is driven by reduced EC proliferative capacity, rather than impaired VEGF-induced migration. Thus, our research challenges the conjecture that aging diminishes ECs’ ability to sense and respond to growth factor signaling. We further observed that reduced proliferation is due to dysfunctional NAD^+^-dependent metabolism, and that supplementation with the NAD^+^ precursor NMN significantly boosts angiogenic capacity in aged mice. Our data strongly support that EC proliferation heavily relies on the mitochondrial TCA cycle to support biomass production and NAD^+^ regeneration. These data may provide new potential therapeutic strategy to counteract NAD^+^ depletion during aging and the age-related decline in tissue perfusion and responsiveness to ischemic diseases.

### Limitations of the study

One of the main limitations of our study is that it was conducted on male mice only. The existence of sex differences in lifespan has been documented in a multitude of species including mice, and we fully acknowledge the importance of investigating this phenomenon in females as well. Furthermore, interventions that extend lifespan in model organisms have demonstrated a sex-specific dimorphism.^81^ However, rodents do not have a menopause phenotype, and the C57BL6/J mouse line has not shown consistent sex differences in the aging process.^82^ In a recent study, it was observed that male mice recapitulated more muscle-aging related pathways characteristic for both male and female humans.^83^ Future studies should incorporate a relevant model of menopausal female mice (e.g., old ovariectomized mice) to provide a comprehensive understanding of female aging and to ensure that the findings are applicable across sexes.

HUVECs are widely used in vascular research due to their key endothelial properties and responsiveness to stimuli like shear stress, cytokines, and growth factors. Their broad use enhances reproducibility across studies, and despite being vein-derived, they share fundamental traits with arterial and microvascular ECs, making them a versatile model. Being of human origin, they also eliminate species-specific differences, increasing the clinical relevance of findings for human health. However, late-passage HUVECs do not exactly mimic the *in vivo* aging process. While we replicated key HUVEC findings in aged lung ECs, the late-passage HUVECs represent a model of replicative senescence that do not match the transcriptional profile of EC isolated from old male mice. While key features of metabolism and the benefits of NMN on EC proliferation and sprouting angiogenesis seem aligned between HUVEC and old mouse EC, future research should aim to validate these findings *in vivo*.

We conducted the ^13^C glucose experiment using only a single time-point, which limits the ability to capture dynamic changes in metabolic flux. A time-course analysis would provide a more comprehensive view of metabolism, enabling the identification of transient metabolic states and the rates of specific reactions. Additionally, incorporating a tracing experiment with ^13^C-labeled glutamine would offer a deeper understanding of alternative metabolic pathways, particularly in the TCA cycle, where glutamine can serve as an important carbon source. This combined approach would provide a more robust and detailed assessment of both glycolytic and glutaminolytic contributions to cellular metabolism, offering insights into pathway interconnectivity and metabolic plasticity.

While promising and generally found safe in clinical trials, NAD precursors may also present some risks. Thus, in certain settings, NMN could promote cancer growth,^84^ and high doses of Nicotinamide have been associated with hepatoxicity in humans.^85^^,^^86^ Furthermore, oral administration of NMN or NR has shown poor bioavailability,^87^^,^^88^ and more work is required to determine the appropriate dose and route of administration of NAD supplementation. Given these limitations, strategies to restore endogenous NAD^+^ levels represent an attractive avenue of research and further studies focused on the kynurenine pathway should be performed.

## Resource availability

### Lead contact

Requests for further information, resources or reagents should be directed to lead contact Alban Longchamp MD, PhD (alongchamp@mgh.harvard.edu).

### Materials availability

This study did not generate any new reagents.

### Data and code availability

• RNA sequencing data have been deposited at NCBI Gene Expression Omnibus (GEO) and are available at https://www.ncbi.nlm.nih.gov/geo/query/acc.cgi?acc=GSE270697.
• Metabolomics data have been deposited at MetaboLights (https://www.ebi.ac.uk/metabolights/studies) and are available under study ID MTBLS11896. All custom scripts are available on github.com at Longchamp-Lab/Kiesworo_et_al_iScience_2024 and of Figshare.com at https://doi.org/10.6084/m9.figshare.28027712.
• Any additional information required to reanalyze the data reported in this paper is available from the lead contact upon request.

## Acknowledgments

This work was supported by the 10.13039/501100001711Swiss National Science Foundation to A.L. (SNSF PZ00P3-185927) and KDB (310030_208041), the Mercier Foundation to A.L., the 10.13039/100008273Novartis Foundation to A.L. and F.A., the 10.13039/501100006387Leenaards Foundation to A.L., the Fondation pour la recherche en chirurgie thoracique et vasculaire to F.A., S.D., and A.L., the Union des Sociétés Suisses des Maladies Vasculaires to S.D.

We are grateful to the Mouse Pathology Facility (MPF), the Cellular Imaging Facility (CIF) of the University of Lausanne, and Miguel Garcia and the Flow Cytometry Core Facility of the EPFL for their support and expertise.

## Author contributions

K.K., M.R.M., K.D.B., J.Z., S.J.M., A.O., F.A., S.D., and A.L. designed the study. K.K., M.L., T.A., M.R.M., S.d’A., G.T., Z.F., A.L., S.J.M., F.A., and A.L. performed the experiments. K.K., T.A., M.R.M., S.M., F.A., S.D., K.D.B., J.Z., T.K., L.R., K.U., H.Y., D.G., and A.L. authored the paper. T.A., A.O., K.D.B., M.R.M., and S.J.M. contributed new reagents or analytic tools. K.K., T.A., M.R.M., K.D.B., J.Z., S.J.M., S.d’A., A.O., F.A., and A.L. participated in data analysis and interpretation. K.D.B., A.O., F.A., S.D., and A.L. obtained funding.

## Declaration of interests

The authors declare no competing interests.

## STAR★Methods

### Key resources table

**Table:** 

REAGENT or RESOURCE	SOURCE	IDENTIFIER
**Antibodies**		

Rabbit anti-Laminin	Sigma	Cat#L9393
Rat Anti-Mouse CD144 (VE-Cadherin)	BD Pharmingen	Cat#555289
Rat Anti-Mouse CD45	BD Biosciences	Cat#550539
Rat Anti Mouse CD68	Biorad	Cat#MCA1957T
Rabbit anti-HO-1	Abcam	Cat#Ab13243
Rabbit anti-ERG	Cell Signaling Technology	Cat#Ab92513
Mouse Anti- BrdU (Clone 3D4)	BD Biosciences	Cat#555627
Rabbit anti-α-Smooth Muscle Actin (D4K9N)	Cell Signaling Technology	Cat#19245
Goat anti-Rabbit Alexa Fluor 680	Thermo Fisher Scientific	Cat#A21109
Goat anti-Rabbit Alexa Fluor 405	Thermo Fisher Scientific	Cat#A31556
Goat anti-Rat Alexa Fluor 488	Thermo Fisher Scientific	Cat#A11006
Donkey anti-Rabbit Alexa Fluor 488	Thermo Fisher Scientific	Cat#A21206
Rabbit anti-Cyclin E1	Cell Signaling Technology	Cat#20808
Rabbit anti-CDK2	Cell Signaling Technology	Cat#2546
Rabbit anti-p21	Cell Signaling Technology	Cat#2947
Rabbit anti-Cyclin D1	Cell Signaling Technology	Cat#2978
Rabbit anti-Phospho-VEGF Receptor 2 (Tyr1175) (19A10)	Cell Signaling Technology	Cat#2478S
Rabbit anti-VEGF Receptor 2 (55B11)	Cell Signaling Technology	Cat#2479
Rabbit anti-Phospho-p44/42 MAPK (Erk1/2) (Thr202/Tyr204)	Cell Signaling Technology	Cat#4370
Rabbit anti-p44/42 MAPK (Erk1/2)	Cell Signaling Technology	Cat#4695
Rabbit anti-Phospho-p38 MAPK (Thr180/Tyr182)	Cell Signaling Technology	Cat#9211
Rabbit anti-p38 MAPK Antibody	Cell Signaling Technology	Cat#9212
Anti-Rabbit HRPO	Thermo Fisher Scientific	Cat#31460
Anti-mouse HRPO	Jackson ImmunoResearch Labs	Cat#115-035-146
anti-mouse CD31 PE antibody (1:400)	BD Biosciences	Cat#553373
anti-mouse CD45 PerCP (1:400)	BD Biosciences	Cat#557235

**Chemicals, peptides, and recombinant proteins**		

β-Nicotinamide mononucleotide (NMN)	Sigma-Aldrich	Cat#N3501
EdU	ThermoFisher Scientific	Cat#A10044
BrdU	Sigma-Aldrich	Cat#B5002
Tripure Isolation Reagent	Roche	Cat#11667165001
Prime Script RT Reagent	Takara	Cat#RR037B
Fast SYBR™ Green Master Mix	ThermoFisher Scientific	Cat#4385618
Dispase II	Sigma-Aldrich	Cat#D4693
Collagenase IV	ThermoFisher Scientific	Cat#17104019
KI8751	Tocris Bioscience	Cat#2542
ZM323881	Sigma-Aldrich	Cat#SML1691
Calcein-AM	ThermoFisher Scientific	Cat#C1430
UK-5099	Sigma-Aldrich	Cat#5048170001
Human VEGF-165 Recombinant Protein	PeproTech®	Cat#100-20-10UG
D-Glucose (U-^13^C_6_)	Cambridge Isotope Laboratories Inc.	Cat#CLM-1396-PK
Corning® Matrigel® Growth Factor Reduced (GFR) Basement Membrane Matrix	BD Biosciences	Cat 356231
Phosphate Buffered Saline (PBS)	Bichsel	Cat#100 0 324
SDS	Promega	Cat# H5114
Tween	Applichem	Cat#A1389
Triton X-100	Sigma-Aldrich	Cat#T8787
Bovine serum AlbuminBSA	Applichem	Cat#A1391
Immobilon Western Chemiluminescent HRP Substate	Millipore	Cat#WBKLS0050
Immobilon-P transfer membrane	Millipore	Cat#IPVH00010
Antifade Mounting Medium with DAPI	Vectashield	Cat#H-1200
Pierce reversible protein Stain Kit for PDGF membranes	Thermofischer Scientific	Cat#24585
Gelatin type B	Sigma-Aldrich	Cat#G9391
Gibco™ Recovery™ Cell Culture Freezing Medium	Thermo Fisher Scientific,	Cat#12648010
EGM-2 Bullet Kit	Lonza	Cat#CC-3162
SYTOX blue (1:1000)	Thermo Fischer Scientific	Cat#S34857
Hanks balanced salt Solution (HBSS)	Gibco	Cat#14170-138
40-μm cell strainer	Corning	Cat#431750
70-μm cell strainer	Corning	Cat#431751
Acrylamide/Bis solution 37:5:1	SERVA	Cat#10681.01

**Critical commercial assays**		

Click-iT™ Plus EdU Cell Proliferation Kit for Imaging, Alexa Fluor™ 594 dye	ThermoFisher Scientific	Cat#C10639
Seahorse XF Mito Stress Test kit	Agilent Technologies	Cat#103015-100
Seahorse XF Glycolysis Stress Test kit	Agilent Technologies	Cat#103020-100
RNeasy Plus Micro Kit	Qiagen	Cat#74034
High Sensitivity RNA ScreenTape System	Agilent	Cat#G2964AA
NAD+/NADH assay kit (colorimetric)	Abcam	Cat#ab65348
Lactate-Glo Assay	Promega	Cat#J5021

**Deposited data**		

RAW RNAseq data	This paper	GSE270697 (GEO Dataset)

**Experimental models: Cell lines**		

Human: Human umbilical vein endothelial cells (HUVECs)	Lonza	Cat#C2519A

**Experimental models: Organisms/strains**		

Mouse: C57BL/6JRj	Janvier Labs	RRID: MGI:2670020

**Oligonucleotides**		

Primer: Arginase (Arg1) Fw: AACCAGCTC TGGGAATCTGC Rv: TCCTGGTACATCT GGGAACTTT	This paper	N/A
Primer: Chitinase-like 3 (Ym1) Fw: AGAA GGGAGTTTCAAACCTGGT Rv: GTCTTG CTCATGTGTGTAAGTGA	This paper	N/A
Primer: Interleukin 10 (IL10) Fw: GCTGTC ATCGATTTCTCCCCT Rv: GACACCTTGG TCTTGGAGCTTAT	This paper	N/A
Primer: Mannose Receptor C-Type 1 (Mrc1) Fw: GAGGCTGATTACGAGCA GTG Rv: TTGGTTCACCGTAAGCCCAAT	This paper	N/A
Primer: Tumor necrosis factor alpha (Tnfα) Fw: ACGGCATGGATCTCAAAG AC Rv: AGATAGCAAATCGGCTGACG	This paper	N/A
Primer: Chemokine (C-C motif) ligand 2 (Ccl2) Fw: CAGGTCCCTGTCATGCTTCT Rv: GCGTTAACTGCATCTGGCTGA	This paper	N/A
Primer: Chemokine (C-X-C motif) ligand 1 (Cxcl1) Fw: CCAGAGCTTGAAGGTGTTGC Rv: CCATTCTTGAGTGTGGCTATGAC	This paper	N/A
Primer: Interleukin 1 beta (IL1b) Fw: GTGTCTGAAGCAGCTATGGCA Rv: CAGGTCATTCTCATCACTGTCAA	This paper	N/A

**Software and algorithms**		

ImageJ (v1.53t)	Fiji (Schneider et al.^89^)	https://imagej.nih.gov/ij/
OmicsPlayground (v2.8.10)	BigOmics Analytics^90^	https://bigomics.ch
MoorLDI Review (v6.1)	Moor Instruments	https://www.moor.co.uk
R (v.4.1.2)	cran.r-project.org	https://cran.r-project.org/bin/windows/base/old/4.1.2/
clusterProfiler (v.4.12.0) package	Wu et al., 2021^91^	https://bioconductor.org/packages/devel/bioc/html/clusterProfiler.html
enrichplot (v.1.24.0) package	Yu G (2024). enrichplot: Visualization of Functional Enrichment Result. https://doi.org/10.18129/B9.bioc.enrichplot	https://bioconductor.org/packages/devel/bioc/html/enrichplot.html
STRING (v11.5)	Szklarczyk et al., 2023^92^	https://string-db.org/
Cytoscape (v3.10)	Cytoscape	https://cytoscape.org/
MetaboAnalyst (v6.0)	Pang et al.^93^	https://www.metaboanalyst.ca/
QuantStudio Design and Analysis (v2.6.0)	ThermoFisher Scientific	https://www.thermofisher.com/ch/en/home/technical-resources/software-downloads/quantstudio-6-7-pro-real-time-pcr-system.html
Seahorse Wave Desktop (v2.6)	Agilent	https://www.agilent.com/en/product/cell-analysis/real-time-cell-metabolic-analysis/xf-software/seahorse-wave-desktop-software-740897
EL-Maven Desktop (v0.12.0)	Elucidata	https://docs.polly.elucidata.io/Apps/Metabolomic%20Data/El-MAVEN.html

### Experimental model and study participant details

#### Mouse strains

3-month (young) and 18-month (aged) old male C57BL/6J mice were used for young/old mice experiments as indicated in the text. All mice were housed at standard housing conditions (22°C, 12 h light/dark cycle), with *ad libitum* access to water and regular diet (SAFE150 SP-25 vegetal diet, SAFE diets, Augy, France). All animal experimentations conformed to the National Research Council: Guide for the Care and Use of Laboratory Animals (National Research Council (U.S.).^94^ All animal care, surgery, and euthanasia procedures were approved by the CHUV and the Cantonal Veterinary Office (SCAV-EXPANIM, authorization number, 3504, 3768).

#### Cell lines

Pooled human umbilical vein endothelial cells (HUVECs; Lonza) were maintained in EGM-2 (Endothelial Cell Growth Medium-2 BulletKit; Lonza) at 37°C, 5% CO_2_ and 5% O_2_ as previously described.^95^ HUVEC were seeded on cell culture plate coated with 1% gelatin type B (Sigma-Aldrich). Early passage HUVECs are defined to be under p10, whereas late passage HUVECs are above p15.

### Method details

#### Hindlimb ischemia (HLI) model

The HLI model was performed as previously described.^73^^,^^96^ Briefly, mice were anesthetized with isoflurane (2.5% under 2.5 L O_2_) and body temperature maintained on a circulating heated pad. Following a 1 cm groin incision, the neurovascular pedicle was visualized under a microscope (Z2 Zoom Stereo Microscope; LW Scientific, Lawrenceville, GA). The femoral nerve and vein were separated from the femoral artery. The femoral artery was ligated proximally, and above both “proximal caudal femoral” and “superficial caudal epigastric” arteries^97^ allowing electrocoagulation of the left common femoral artery while sparing the vein and nerve. Buprenorphine (0.1 mg/kg Temgesic, Reckitt Benckiser AG, Switzerland) was provided before surgery, as well as a post-operative analgesic every 12h for 36 h. Mice were euthanized under anesthesia by cervical dislocation and exsanguination at time points indicated. Muscles were either frozen in OCT for histology, or flash frozen directly in liquid nitrogen for molecular analyses.

To measure cell proliferation *in vivo*, EdU (A10044, ThermoFisher Scientific) was diluted in NaCl at a concentration of 2 mg/mL and 500 μg was injected via i.p. injection 16h before sacrifice. Mice were sacrificed at day 5 post-HLI; ischemic muscles were placed in OCT and frozen in liquid nitrogen vapor.

#### Laser Doppler perfusion imaging (LDPI)

Laser Doppler perfusion imaging (LDPI) was performed as described previously.^73^^,^^96^ Briefly, mice were kept under isoflurane anesthesia, and body temperature maintained on a circulating heated pad. Once unconscious, we subjected the mouse hindlimbs to the LDPI (moorLDI2-HIR; Moor Instruments) system with a low-intensity (2 mW) laser light beam (wavelength 632.8 nm). Hindlimb blood flow was recorded as a 2D color-coded image, with a scan setting of 2 m/pixel. Blood flow recovery was monitored at baseline, d0 (immediately post-surgery), d1, d3, d5, d7, d10, and d14. LDPI intensity of the ischemic foot was normalized to the corresponding contralateral foot and expressed as ratio between the ischemic/non-ischemic limb.

#### Immunohistochemistry (IHC)

IHC was performed on 10 μm frozen sections of gastrocnemius muscle. After 5 min fixation in PFA 4% and rinsing in PBS, immunostaining was performed as previously described.^73^^,^^96^ Muscle sections were permeabilized in PBS supplemented with 2 wt. % BSA and 0.1 vol. % Triton X-100 for 30 min, blocked in PBS supplemented with 2 wt. % BSA and 0.1 vol. % Tween 20 for another 30 min, and incubated overnight in the primary antibody diluted in the same buffer. Subsequently, the slides were washed three times for 5 min in PBS supplemented with 0.1 vol. % Tween 20, and incubated for 1 h at room temperature with a mix of appropriate fluorescent-labeled secondary antibodies. The antibodies used are described in the key resources table. The slides were then washed and mounted in DAPI-containing Vectashield fluorescent mounting medium. EdU immunostaining was performed according to the manufacturer’s instructions (Click-iT Plus EdU Cell Proliferation Kit for Imaging, Alexa Fluor 594 dye, ThermoFischer). Sections were then scanned with a Zeiss Axioscan microscope. VE-cad positive area of whole muscle was quantified blindly using Fiji software (ver. 1.53t; http://fiji.sc/Fiji). Quantifications were expressed as a percentage of VE-cad-positive area to the total surface area of the gastrocnemius muscle.

#### Reverse transcription and quantitative polymerase chain reaction (RT-qPCR)

Pulverized frozen gastrocnemius muscles were homogenized in Tripure Isolation Reagent (Roche, Switzerland), and total RNA was extracted as published.^73^ After RNA Reverse transcription (Prime Script RT reagent Cat#RR037B, Takara), cDNA levels were measured by qPCR Fast SYBR Green Master Mix (Cat#4385618, Applied Biosystems, ThermoFisher Scientific AG, Switzerland) in a Quant Studio 5 Real-Time PCR System (Applied Biosystems, ThermoFischer Scientific AG, Switzerland). DNA oligo primers used in our analyses are described in the key resources table.

#### Blood sampling

Peripheral blood was collected from the mice tail vein into EDTA tubes, pre-operatively and at 2 days post-HLI. Plasma was harvested and frozen at −80°C. Leukocyte and erythrocyte fraction were then treated with 10mL of erythrocyte lysis buffer (0.15M NH_4_Cl, 5.7x10^−3^ KH_2_PO_4_, 1x10^−4^ Na_2_EDTA) for 5 min. Leukocytes were then stored at −80°C in Gibco Recovery Cell Culture Freezing Medium (12648010, Thermo Fisher Scientific, Zurich, Switzerland).

#### CyTOF

All samples were barcoded and processed simultaneously for antibody staining (markers selected: CD45, CD3, CD4, CD8, CD11b, CD11c, CD19, CD24, CD25, CD44, CD62L, CD64, CD103, CD117, F4/80, Ly6C, Ly6G/C, MHCII, NK1.1, SiglecF, TCRb, FoxP3, B220, CD69, CD127, Tbet, KLRG1; these prevalidated and pretitrated metal-conjugated antibodies were purchased from Fluidigm (Standard BioTools)). The sample was mixed and incubated for 30 min at room temperature. After incubation, the sample was washed twice with Maxpar Cell Staining Buffer. Cells were then fixed by incubating the sample with 1 mL of 1.6% paraformaldehyde for 10 min. Subsequently, cells were washed twice with Maxpar Perm-S Buffer and centrifuged for 10 min at 1000g. Cells were then resuspended in 400 μL of Maxpar Perm-S Buffer and incubated for other 30 min with cytoplasmic/secreted antibody cocktail (1:100 dilution for each antibody, final volume 800 μL). At the end of the incubation, cells were washed twice with Maxpar Cell Staining Buffer and stained overnight with Cell-ID Intercalator-Ir solution at the final concentration of 125 × 10−9 m. Prior to data acquisition, the samples were washed twice with Maxpar Cell Staining Buffer, resuspended with 2 mL of Maxpar water and filtered using a 0.22 μm cell strainer cap to remove possible cell clusters or aggregates.

Stained cells were analyzed on a CyTOF2/Helios mass cytometer (Fluidigm, San Francisco, USA) at an event rate of 400–500 cells per second. Data files for each barcoded sample were concatenated using an in-house script. The data were normalized using Normalizer v0.1 MCR. Files were debarcoded using the MATLAB Debarcoder Tool.

#### Tissue EC isolation

Mice were euthanized and leg muscles or lungs were immediately dissected and placed in a Petri dish on ice. Tissues were minced with a scalpel until a homogeneous paste-like mash was formed. Subsequently, the tissue was enzymatically digested in a buffer containing 2 mg/mL Dispase II (D4693, Sigma-Aldrich, Steinheim, Germany), 2 mg/mL Collagenase IV (17104019, ThermoFisher Scientific, Zurich, Switzerland), 2 mM CaCl2 and 2% BSA in PBS at 37°C for 10 min (lungs) or 25 min (muscle), with gentle shaking every 3 min. The reaction was stopped by immediately adding an equal volume of ice-cold HBSS containing 20% FBS and the suspension was passed through a 70-μm cell strainer (#431751, Corning, New York, USA) then 40-μm cell strainer (#431750, Corning, New York, USA) to remove tissue debris. Cell suspension was centrifuged at 500 g for 5 min at 4°C, then the pellet was washed with ice-cold HBSS (+20% FBS) followed by a centrifugation at 400*g* for 5 min in 4°C. For cell culture experiments, ECs were selected with magnetic beads as performed according to the manufacturer’s protocol (CD31 MicroBeads, 130-097-418, Miltenyi Biotec, Bergisch Gladbach, Germany). Isolated mouse ECs were seeded on cell culture plate coated with 1% gelatin type B (Sigma-Aldrich), and maintained in EGM-2 at 37°C, 5% CO_2_ and 5% O_2_.

For bulk RNASeq, EC selection was performed by flow cytometry. The cell pellet was re-suspended in antibody medium (EGM2 CC-3162, Lonza, Basel, Switzerland) with anti-mouse CD31 PE antibody (1:400) (553373, BD Biosciences, Basel, Switzerland) and anti-mouse CD45 PerCP antibody (1:400) (557235 BD Biosciences, Basel, Switzerland) and placed on ice for 20 min in the dark. Before sorting, the cell suspension was washed in FACS buffer (1xPBS+1%BSA) and centrifuged at 400G for 5 min, 4°C, then the washed cell pellet was re-suspended in FACS buffer containing cell viability dye, SYTOX blue (1:1000) (S34857, Thermo Fischer Scientific, Zurich, Switzerland). Viable ECs (CD31^+^, CD45^−^, SYTOX blue-) were sorted by a FACS Aria III (BD Bioscience) sorter. ECs were put in culture in EGM2 cell culture medium (CC-3162, Lonza, Basel, Switzerland) or directly sorted (70μm nozzle) into 700 μL RNA lysis buffer for RNA extraction using RNeasy Plus Micro Kit (74034 QIAGEN).

#### Bulk RNASeq

RNA extraction from sorted primary mouse EC was performed using RNeasy Plus Micro Kit (74034 QIAGEN). RNA quality test was performed by Agilent High Sensitivity RNA ScreenTape System (G2964AA). Samples with RNA Integrity Number (RIN)≥8.0 were further analyzed. Bulk RNA-seq libraries were obtained following the Smartseq II recommended protocol. Libraries were sequenced on the Novaseq 6000 (Illumina) instrument and sequenced data were processed using Kallisto to generate a count file matrix for each individual sample. Samples were pooled together on a single dataset for downstream analysis and genes with one count or less across all samples were filtered out.

#### Analysis of bulk RNASeq

The bioinformatics platform OmicsPlayground v2.8.10 (BigOmics Analytics; Lugano, Switzerland) was used for data pre-processing, statistical computation and visualization.^90^ Data pre-processing included filtering of genes based on variance, the expression across the samples, and the number of missing values. Only protein coding genes on non-sexual chromosomes have been kept. A tSNE plot was generated to visualise the similarity in the gene expression profile of individual samples. For gene-level testing and identification of differentially expressed genes (DEG), statistical significance was assessed using two independent statistical methods: Voom and Limma-no-trend. The maximum q-value of the two methods was taken as aggregate q-value, which corresponds to taking the intersection of significant genes. A volcano plot was generated to visualise these genes.

Gene expression have been normalized using logCPM normalization in the edgeR R/Bioconductor package. All the 500 DEGs were analyzed for functional enrichment of GO terms and pathways using STRING v11.5^92^ (Search Tool for the Retrieval of Interacting Genes), where only high interaction score network edges were considered (>0.7 confidence score). To identify the most relevant clustering modules in the Protein–Protein Interactions network, we subclustered the network using the Markov Cluster Algorithm (MCL) with an infiltration of 1.8, in Cytoscape. Singletons were excluded and the different clusters were then colored. Geneset enrichment analysis (GSEA) was performed in R (v.4.1.2) using clusterProfiler (v.4.12.0),^91^ and enrichplot (v.1.24.0) packages. The 3 following databases were used for enrichment: GO Biological process (BP), KEGG pathway and Reactome pathway. The *p*-values were adjusted using Benjamini-Hochberg method and terms with a False Discovery Rate (FDR) < 0.05 were collected. The clusters without relevant pathway enrichment or non-interacting genes were ignored. Chord diagram with top pathways for each have been performed using GOplot 1.0.2 package (https://cran.r-project.org/web/packages/GOplot/index.html). Computational metabolic pathway variation was evaluated as previously described (https://github.com/LocasaleLab/Single-Cell-Metabolic-Landscape).^45^ Lists of metabolic genes and pathways were obtained from the KEGG database (http://www.kegg.jp).

#### Aortic ring sprouting assay

Aortic ring assay was previously described. Briefly, mouse thoracic aortas were isolated and cut into 1 mm-wide rings and embedded in Corning Matrigel Growth Factor Reduced Basement Membrane Matrix (BD Biosciences Cat#356231) and incubated in full EGM2 medium (Lonza) at 37°C, 5% CO_2_ and 5% O_2_. For the NMN supplementation experiments (β-Nicotinamide mononucleotide; Sigma-Aldrich; N3501), 0.5 and 2mM concentration was used as indicated. Media was replaced every three days. Brightfield images was taken at 2X using a Nikon TI-2 live-cell imaging microscope (six sprouts/mouse aorta). Length of sprouts originating from the aorta were quantified using the Fiji software (ImageJ 1.53t).

#### VEGF inhibition

To understand the contribution of VEGF on proliferation and migration, we incubated HUVECs with the VEGF inhibitors KI8751 (Tocris Bioscience; 100 nM) and ZM323881 (Sigma-Aldrich; 2 μM) for the duration of the assay.

#### Cell migration assay

HUVEC were grown to confluence in a 12-well plate and a scratch wound was created using a sterile p200 pipette tip as previously described.^73^^,^^96^ Repopulation of the wound in presence of Mitomycin C was recorded by phase-contrast microscopy over 16 h in a Nikon Ti2-E live-cell imaging microscope. The denuded area was measured at t = 0h and t = 10h after the wound and quantified using the ImageJ software (ver. 1.53t; http://fiji.sc/Fiji). Data were expressed as a ratio of the healed area over the initial wound area.

#### Cell transmigration assay

The Boyden chamber assay was used to investigate the ability of ECs to transmigrate across a matrix barrier toward a chemotactic stimulus. This was performed using a 24-well tissue culture plates with Fluoroblok inserts containing 8-μm pore-size polycarbonate membrane (Corning, New York, USA). HUVECs were trypsinized and resuspended in EBM-2 (Endothelial Cell Growth Basal Medium-2, Lonza). 500 μL EGM-2 BulletKit culture medium (with full supplements) was added into the bottom wells. ECs were subsequently loaded into the upper wells (10^5^ cells in 500 μL). After 8 h in cell culture, Calcein-AM (5 μg/mL, ThermoFisher Scientific) was added to the well to stain the cells on the outer surface of the membrane of the transwell. After 30 min and two washes with PBS, fluorescence was measured using a fluorescent plate reader (Synergy H1, BioTek AG; λex = 485 nm; λem = 530 nm).

#### Cell proliferation assay

HUVEC were grown at 80% confluence (5x10^3^ cells per well) on glass coverslips in a 24-well plate and starved overnight in serum-free medium (EBM-2, Lonza). They were then incubated for 6 h in EGM2 containing 10μM BrdU. Immunostaining was performed on cells washed and fixed for 5 min in −20°C methanol, air-dried, rinsed in PBS and permeabilized for 10 min in PBS supplemented with 2% BSA and 0.1% Triton X-100. BrdU positive nuclei were automatically detected in ImageJ software (ver. 1.53f51; http://fiji.sc/Fiji) and normalized to the total number of DAPI-positive nuclei.^96^

#### Flow cytometry (cell cycle assessment)

HUVECs were grown at 70% confluence (5x10^4^ cells per well), before trypsinization and collection. Subsequently, the HUVECs were washed in ice-cold PBS before fixation by dropwise addition of ice-cold 70% ethanol while slowly vortexing the cell pellet. Cells were fixed for 1 h at 4°C, washed 3 times in ice-cold PBS and resuspended in PBS supplemented with 20 μg/mL RNAse A and 10 μg/mL DAPI. Flow cytometry was performed in a Cytoflex-S apparatus (Beckman Coulter GmbH, Krefeld, Germany).

#### Seahorse

Glycolysis and Mitochondrial stress tests were performed on confluent HUVECs or primary mouse lung EC according to the manufacturer’s kits and protocols (Agilent Seahorse XF glycolysis stress test kit, Agilent Technologies, Inc.). 1 μM Oligomycin was used. Data were analyzed using the Seahorse Wave Desktop Software (Agilent Technologies, Inc., Seahorse Bioscience).

#### NAD^+^/NADH quantification

The NAD^+^/NADH level was determined using the NAD^+^/NADH assay kit (Abcam, Cambridge, UK) according to the manufacturer’s instructions as previously described.^28^ HUVECs were grown at 70% confluence (5x10^4^ cells per well) and treated with 2mM NMN for 24h. Cells were collected directly in the assay buffer from the kit as suggested by the manufacturer and normalized to the protein content using the Pierce BCA protein assay kit (Thermo Fisher Scientific, Waltham, MA), with 250 μg of protein used for each reaction.

#### Lactate quantification

HUVECs were grown at 70% confluence (5x10^4^ cells per well) and treated as indicated for 24h in full EGM2 cell culture medium. Cell supernatants were collected and immediately flash frozen in liquid nitrogen. Lactate was quantified using the Lactate-Glo Assay (Cat J5021; Promega AG, Switzerland) according to the manufacturer’s instructions. 50μL of supernatant was combined with 50μL of Lactate Detection Reagent into a 96-well assay plate, incubated 60 min at room temperature in the dark before reading luminescence in a multimodal plate reader (Synergy H1, BioTek AG).

#### Senescence-associated beta galactosidase staining

HUVECs were cultured on glass cover slips at 80% confluence. Cells were washed with PBS before fixing with neutral buffered 4% PFA for 3 min at room temperature. After another wash, fixed cells were incubated for 12-16 h at 37^o^C with staining solution for beta galactosidase (1 mg/ml X-gal, 1X citric acid/sodium phosphate buffer pH 6.0, 5mM potassium ferricyanide, 5mM potassium ferrocyanide, 150mM NaCl, 2mM MgCl_2_). Subsequently, the coverslips were then washed once before mounting with 50% glycerol on microscope slides. Images were taken on Nikon TS100 light microscope.

#### ^13^C_6_ glucose tracing and metabolite extraction

HUVEC were grown at 80% confluence (20.10^3^ cells per well) in a 6-well plate and starved overnight in serum-free medium (EBM-2, Lonza). Then, medium was replaced with 2 mL/well of DMEM media containing D-Glucose (U-^13^C_6_, CLM-1396-PK, Cambridge Isotope Laboratories Inc.) 3 h prior to collection. Prior to harvest, labeled media was removed, and HUVECs were washed with PBS. Metabolite extraction from HUVECs was performed as rapidly as possible to minimize perturbations in metabolism. Cell metabolism was quenched, and metabolites were extracted by quickly aspirating media from culture dishes and adding cold (−20°C) 80:20 methanol/water. Then, cells were scraped off the culture dish surface, transferred to an Eppendorf tube and centrifuged at 4C to remove precipitate. The supernatant was collected for analysis by LC-MS. Note that, in all cases, quenching was performed without any washing steps that can perturb the metabolism.

#### LC-MS metabolomics analysis

^13^C_6_ glucose HUVEC samples were analyzed by hydrophobic interaction chromatography (HILIC) coupled with a quadrupole-orbitrap mass spectrometer (Thermo Scientific Exploris 480). Separation on LC used an XBridge BEH Amide column (150 mm × 2.1 mm with 2.5uM particle size) with solvent A (95:5 acetonitrile: water with 20mM ammonium acetate, 20mM ammonium hydroxide, pH 9.4) and solvent B (100% acetonitrile). The gradient used was 0 min, 90%B; 3 min, 75% B; 8 min, 70% B; 10 min, 50% B; 13 min, 25% B; 16 min, 0% B; 21 min, 90%B. The flow rate was 150uL per minute with a 10uL injection volume. The column temperature was 25C. The MS full scans were in dual mode with negative window 70-600m/z and positive window 120–900 m/z. Raw files were converted to mzXML format using msConvert and peaks were picked using a validated platform-specific knowns list in EL-MAVEN software (Elucidata). C13 natural isotope abundance was corrected using Iso-Autocorr in MATLAB (https://github.com/xxing9703/Iso-Autocorr). Downstream analyses were performed in R and MetaboAnalyst 6.0 (https://www.metaboanalyst.ca/). Metabolite abundance was normalized by protein content and log transformed. The multivariate analysis was an unsupervised principal component analysis (PCA), which was followed by univariate analysis including false discovery rate calculated by the method of Benjamini, Hochberg, and Yekutieli and fold change (FC) analysis. Differential metabolites were defined as metabolites with a q value >0.05, and a fold change >1.4. Quantitative Enrichment Analysis (QEA) of differential metabolites was conducted using the Kyoto Encyclopedia of Genes and Genomes (KEGG) database. Heatmapping was used to better show the trend changes and internal differences of different metabolites.

### Quantification and statistical analysis

All experiments adhered to the ARRIVE guidelines and followed strict randomization. All experiments and data analysis were conducted in a blind manner using coded tags rather than actual group name. A power analysis was performed prior to the study to estimate sample-size. We hypothesized that age would reduce neovascularization by 50%. Using an SD at +/− 20% for the surgery and considering a power at 0.8, we calculated that *n* = 6 to 8 animals/group was necessary to validate a significant effect. Animals with pre-existing conditions (malocclusion, injury, abnormal weight, tumors) were not operated or excluded from the experiments upon discovery during dissection. All experiments were analyzed using GraphPad Prism 10. Normal distribution of the data was assessed using Kolmogorov-Smirnov tests. All data with a normal distribution were analyzed by paired or unpaired bilateral Student’s t-tests, or Matched Mixed-effects model (REML) followed by multiple comparisons using post-hoc t-tests with the appropriate correction for multiple comparisons. For non-normally distributed data, Kruskal-Wallis non-parametric ranking tests were performed, followed by Dunn’s multiple comparisons test to calculate adjusted *p* values. Data are displayed as means ± standard deviation (S.D.). Unless otherwise specified, *p*-values are reported according to the APA 7^th^ edition statistical guidelines. ∗*p* <0 .05, ∗∗*p* <0 .01, ∗∗∗*p* <0 .001.

## Supplemental information

Document S1. Figures S1–S5 and Tables S1 and S2

Table S3. Pathway enrichment analysis
