# Supplementary material for: Nicotinamide mononucleotide restores impaired metabolism, endothelial cell proliferation and angiogenesis in old sedentary male mice
Source: iScience. 2024 Dec 20;28(1):111656. doi: 10.1016/j.isci.2024.111656 (PMC11763620; doi:10.1016/j.isci.2024.111656)
Supplement: Document S1. Figures S1–S5 and Tables S1 and S2 [file mmc1.pdf]

iScience, Volume *28*

Supplemental information

Nicotinamide mononucleotide restores impaired metabolism, endothelial cell proliferation and angiogenesis in old sedentary male mice

Kevin Kiesworo, Thomas Agius, Michael R. Macarthur, Martine Lambelet, Arnaud Lyon, Jing Zhang, Guillermo Turiel, Zheng Fan, Sènan d'Almeida, Korkut Uygun, Heidi Yeh, Sébastien Déglise, Katrien de Bock, Sarah J. Mitchell, Alejandro Ocampo, Florent Allagnat, and Alban Longchamp

Supplemental Table S1: Antibodies

Target antigen	Vendor	Catalog #	Working concentration
Laminin	Sigma	L9393	1/200 (IHC)
VE-Cadherin	BD Pharmingen	555289	1/200 (IHC)
CD45	BD Biosciences	550539	1:200
CD68	Biorad	MCA1957T	1/500 (IHC)
HO-1	Abcam	13243	1/200 (IHC)
ERG	Cell Signaling Technology	#4695	1/100 (IHC)
BrdU	BD Biosciences	555627	1/200 (ICC)
α-SMA	Cell Signaling Technology	#19245	1/500 (IHC)
Goat anti-Rabbit Alexa Fluor 680	Thermo Fisher Scientific	A21109	1/500 (IHC)
Goat anti-Rabbit Alexa Fluor 405	Thermo Fisher Scientific	A31556	1/500 (IHC)
Goat anti-Rat Alexa Fluor 488	Thermo Fisher Scientific	A11006	1/500 (IHC)
Donkey anti-Rabbit Alexa Fluor 488	Thermo Fisher Scientific	A21206	1/500 (IHC)
Cyclin E1	Cell Signaling Technology	20808	1/1000 (WB)
CDK2	Cell Signaling Technology	2546	1/1000 (WB)
p21	Cell Signaling Technology	2947	1/1000 (WB)
Cyclin D1	Cell Signaling Technology	2978	1/1000 (WB)
pVEGFR2	Cell Signaling Technology	2478S	1/1000 (WB)
VEGFR2	Cell Signaling Technology	2479	1/1000 (WB)
pERK	Cell Signaling Technology	4370	1/2000 (WB)
ERK	Cell Signaling Technology	4695	1/1000 (WB)
p-p38	Cell Signaling Technology	9211	1/1000 (WB)
p38	Cell Signaling Technology	9212	1/1000 (WB)
Anti-Rabbit HRPO	Thermo Fisher Scientific	31460	1/20000 (WB)
Anti-mouse HRPO	Jackson ImmunoResearch Labs	115-035-146	1/15000 (WB)

Detailed information is available in the **STAR METHODS Key Resources Table**

Supplemental table S2: DNA oligo primers

Primer Name	Gene	Sequence
Arg1	Arginase	Fw: AACCAGCTCTGGGAATCTGC<br>Rv: TCCTGGTACATCTGGGAACTTT
Ym1	Chitinase-like 3	Fw: AGAAGGGAGTTTCAAACCTGGT<br>Rv: GTCTTGCTCATGTGTGTAAGTGA
IL10	Interleukin 10	Fw: GCTGTCATCGATTTCTCCCCT<br>Rv: GACACCTTGGTCTTGGAGCTTAT
Mrc1	Mannose Receptor C-Type 1	Fw: GAGGCTGATTACGAGCAGTG<br>Rv: TTGGTTCACCGTAAGCCCAAT
Tnfα	Tumor necrosis factor alpha	Fw: ACGGCATGGATCTCAAAGAC<br>Rv: AGATAGCAAATCGGCTGACG
Ccl2	Chemokine (C-C motif) ligand 2	Fw: CAGGTCCCTGTCATGCTTCT<br>Rv: GCGTTAACTGCATCTGGCTGA
Cxcl1	Chemokine (C-X-C motif) ligand 1	Fw: CCAGAGCTTGAAGGTGTTGC<br>Rv: CCATTCTTGAGTGTGGCTATGAC
IL1b	Interleukin 1 beta	Fw: GTGTCTGAAGCAGCTATGGCA<br>Rv: CAGGTCATTCTCATCACTGTCAA

Detailed information is available in the **STAR METHODS Key Resources Table**

Supplemental Figures and Legends

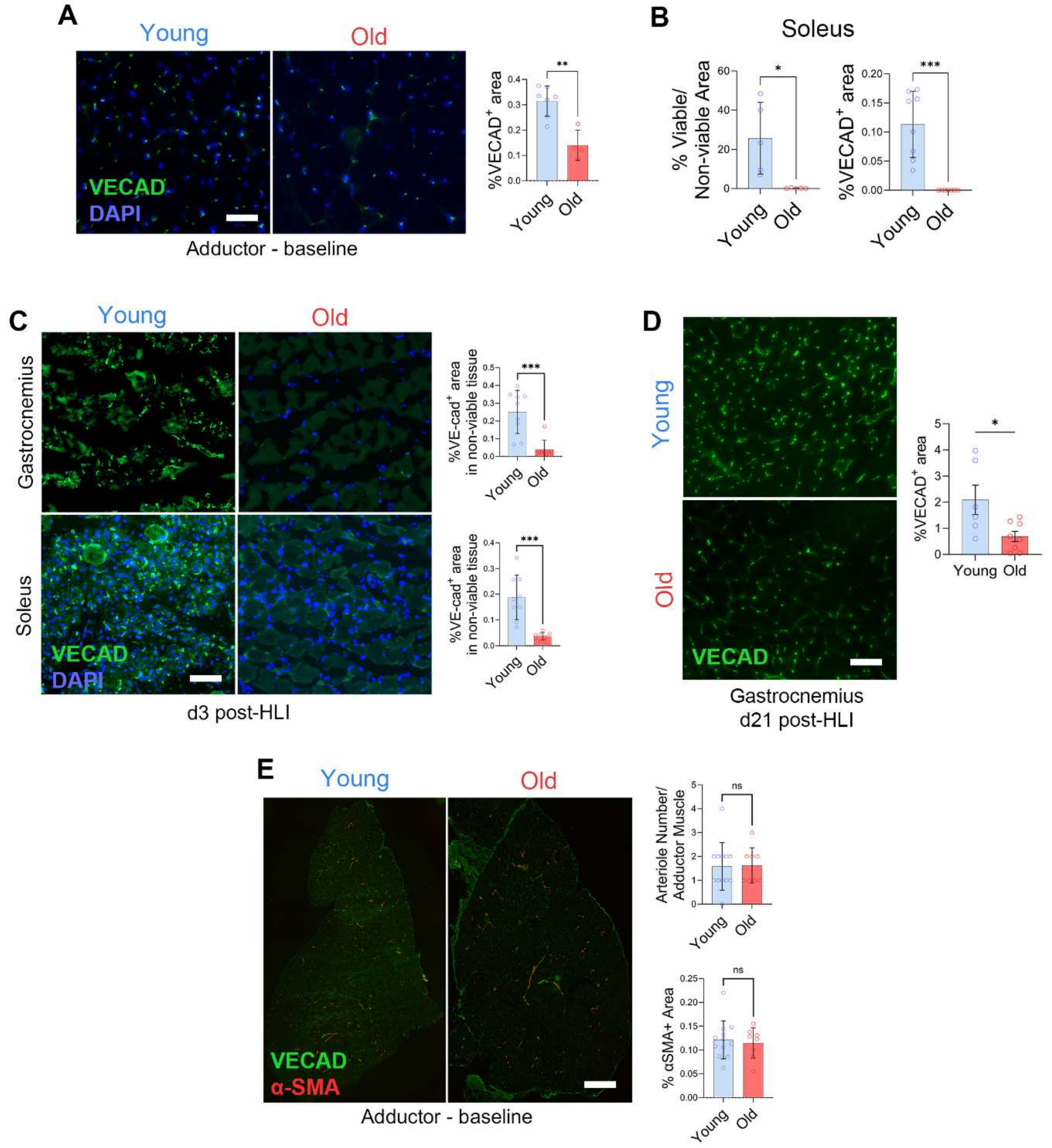

Fig. S1: Aged mice undergo slower neovascularisation and suffer more muscle damage post-hindlimb ischemia.

(A) Adductor muscle microvasculature at baseline as depicted by representative images (left) and quantification (right) of transverse sections stained with VE-cad. n=8-12 per group. Scale bar represents 50µm. **(B)** Quantification of ischemic soleus muscle microvasculature at 3 days post-HLI of transverse sections stained with VE-cad. n=8-9 per group. Scale bar represents 50µm. **(C)** Microvasculature within the ischemic regions of gastrocnemius and soleus muscle at 3 days post-HLI as depicted by representative images (left) and quantification (right) of transverse sections stained with VE-cad. n=8-9 per group. Scale bar represents 50µm. **(D)** Quantification of gastrocnemius muscle microvasculature at 21 days post-HLI of transverse sections stained with VE-cad. n=8-9 per group. Scale bar represents 150µm. **(E)** Arterioles in baseline adductor muscle as depicted by representative images (left) and quantification (right) of transverse sections stained with VE-cad and α-SMA. n=8-12 per group. Scale bar represents 800µm. Data are expressed as mean ± S.D. ** p≤.01, *** p≤.001 and **** p≤.0001 by bilateral unpaired Student's t-test. See also main figures 1 and 2.

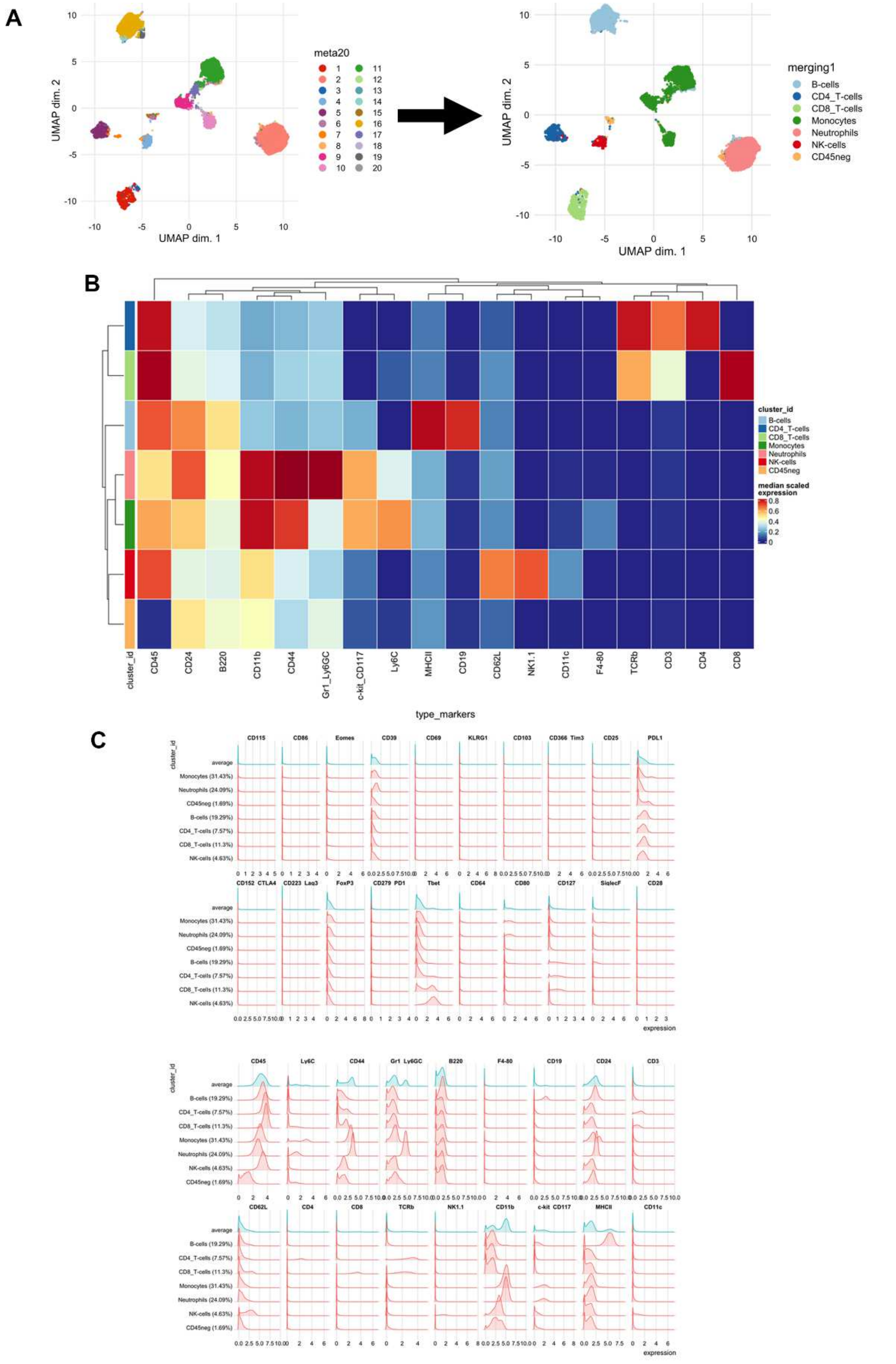

Fig. S2: CyTOF analysis on immune recruitment post-hindlimb ischemia in young and old mice.

(A) UMAP plot for the PBMC dataset, where cells are coloured according to the manual merging of the 20 cell populations, obtained with FlowSOM, into 7 PBMC populations. **(B)** Heatmap of the median marker intensities of the 7 PBMC populations obtained with FlowSOM. **(C)** Distributions of marker intensities (arcsinh-transformed) of the 7 PBMC populations obtained with FlowSOM. See also main figure 2.

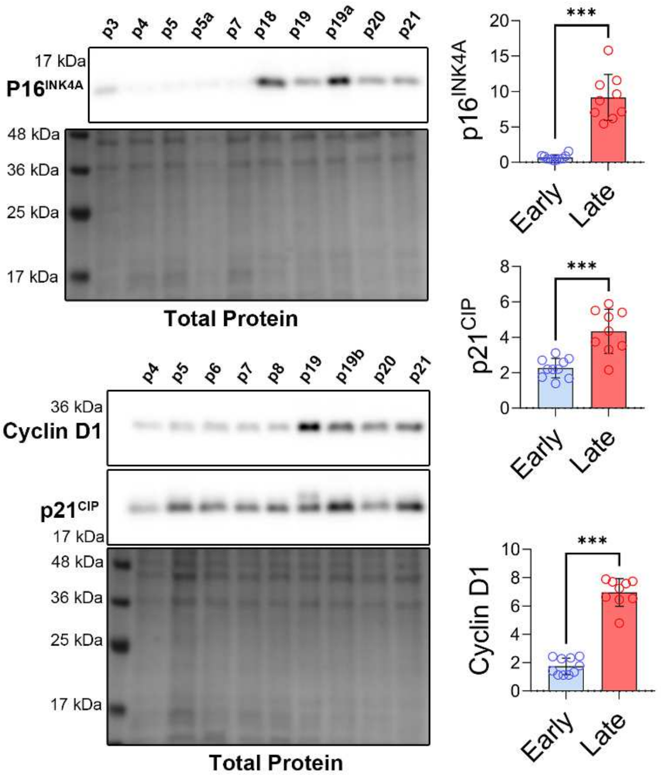

Fig. S3: Impairment of EC proliferation results in neovascularisation defect in old mice
Western blot of p16^{INK4A}, p21^{CIP} and Cyclin D1 on lysates of early and late passage HUVECs, as indicated. All proteins normalized to total protein stain. Data are expressed as mean ± S.D. *** p≤.001 by bilateral unpaired Student’s t-test. See also main figure 3

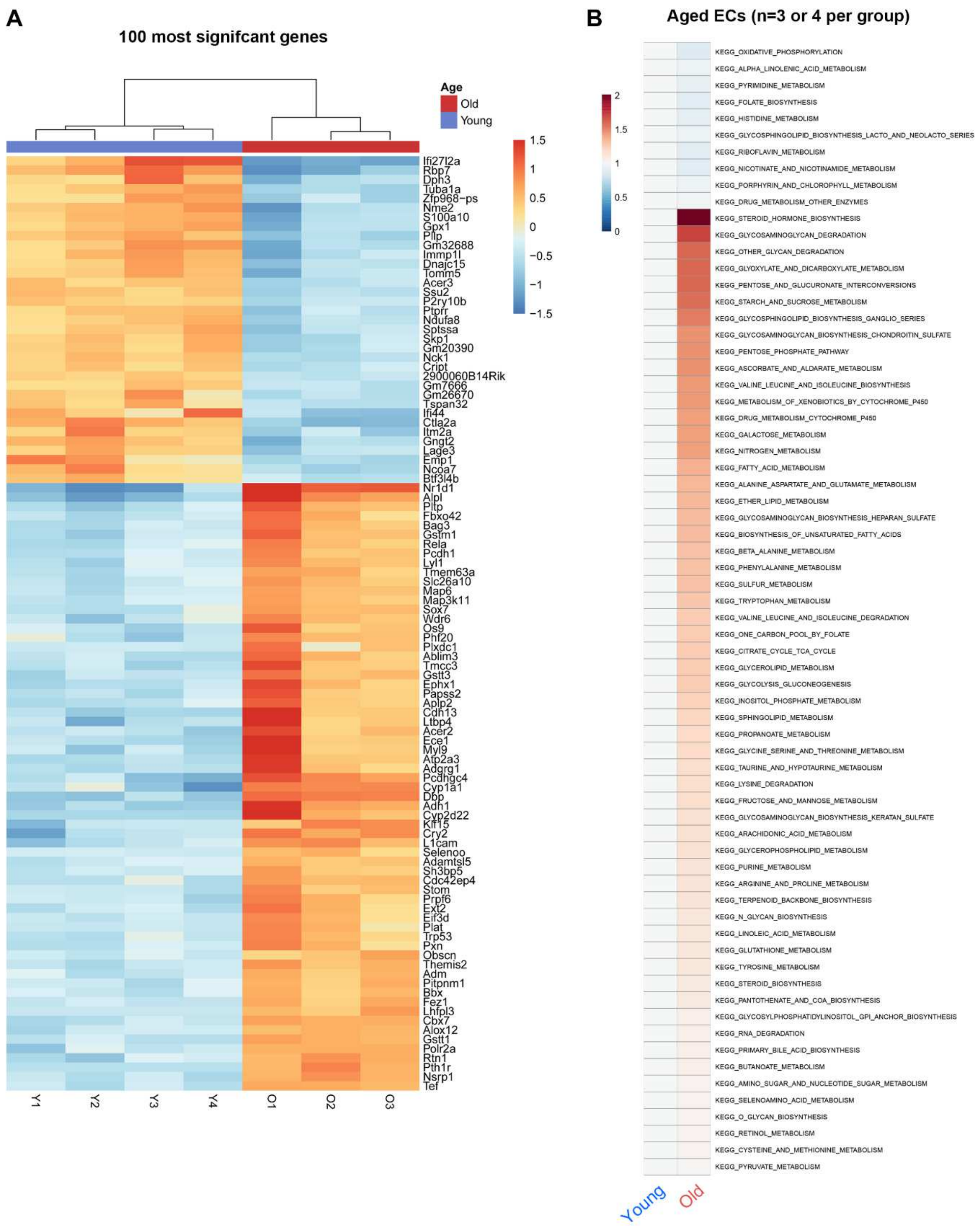

Fig. S4: Old ECs exhibit an altered metabolic gene expression profile.
(A) Heatmap representing the expression of the top 100 most significant genes in ECs harvested from young and old mice gastrocnemius muscle n=3-4 per group. **(B)** Enrichment of metabolism-associated KEGG pathways in ECs harvested from young and old mice. n=3-4 per group. See also main figure 4.

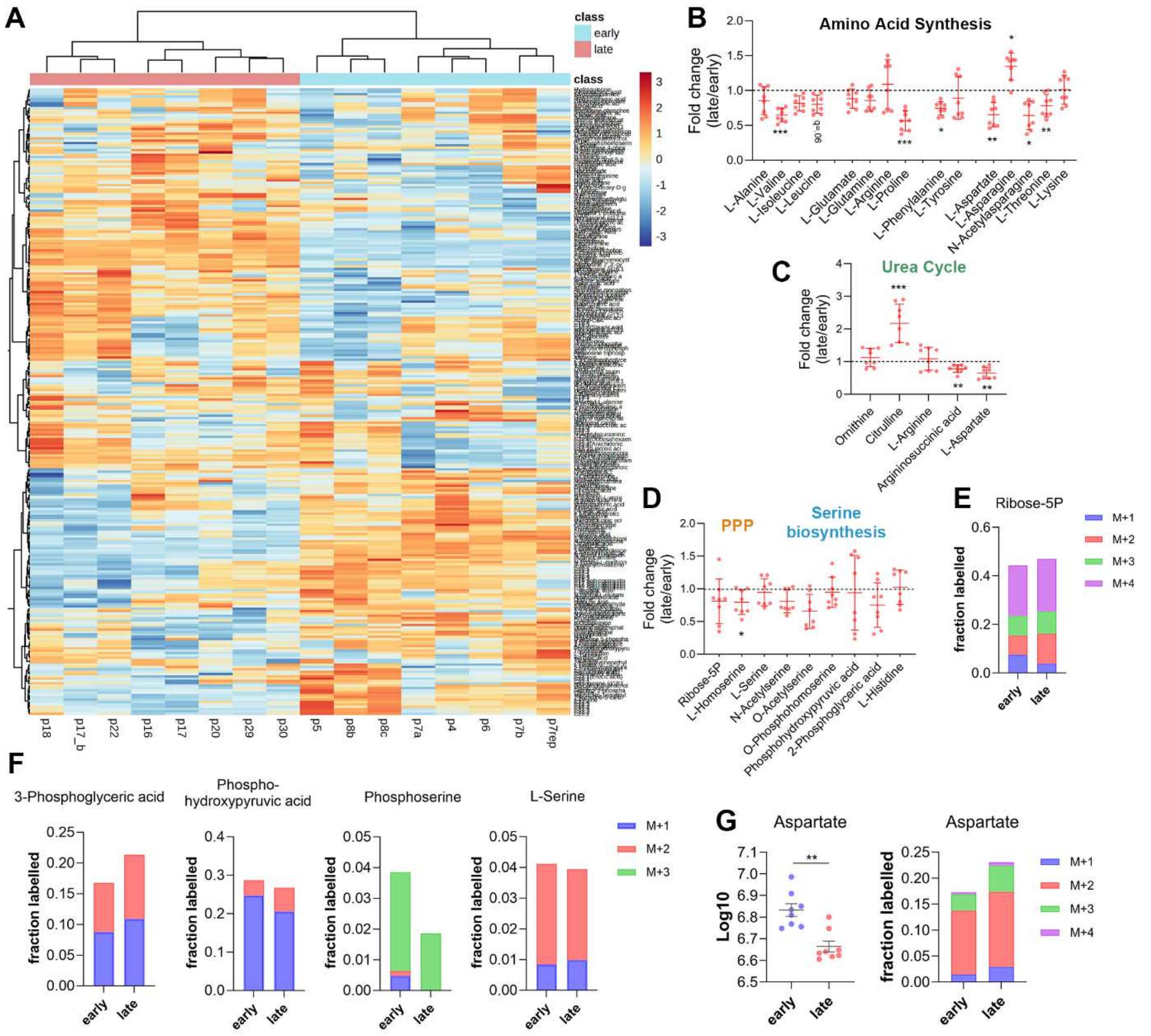

Fig. S5: Late passage HUVECs exhibit vast changes in metabolism

(A) Unbiased k-means clustering of polar metabolites from early- and late-passage HUVECs. **(B)** Relative abundance of individual metabolites expressed as fold change in the pentose phosphate and serine biosynthesis pathways. **(C-D)** Glucose-derived ^{13}C enrichment into intermediates within the (**C**) pentose phosphate and (**D**) serine biosynthesis pathways. **(E)** Relative abundance of aspartate (left). Glucose-derived ^{13}C enrichment into aspartate. Statistical significance determined through false discovery rate calculated by the method of Benjamini, Hochberg, and Yekutieli; ** q<.01. n=8 per group. B-D) Data are presented with mean ± S.D. See also main figure 5.
